# Mechanical Non-Equivalence of Imposed-Strain Representations in Bond–Slip Modelling of Restrained RC Ties

**DOI:** 10.3390/ma19214460

**Published:** 2026-10-23

**Authors:** Mariusz Zych

**Affiliations:** Faculty of Civil Engineering and Transport, Cracow University of Technology, 31-155 Cracow, Poland; mariusz.zych@pk.edu.pl

**Keywords:** bond–slip, crack width, imposed strain, restrained reinforced concrete, tension stiffening, finite-element modelling, transfer length

## Abstract

**Highlights:**

- Nominally equivalent imposed-strain models are not mechanically equivalent.
- Loading representation strongly affects reinforcement-stress histories.
- Final crack widths alone cannot validate an imposed-strain model.
- End-displacement modelling best reproduced the corresponding tie tests.
- Evolving transfer lengths govern stepwise crack formation and crack widths.

**Abstract:**

Imposed deformations caused by shrinkage and temperature are a major source of early-age and serviceability cracking in restrained reinforced concrete (RC) members, including tanks, water-retaining structures and containment structures. A bond–slip-based finite-element framework is presented for simulating crack initiation, stepwise crack formation, crack-width evolution and reinforcement-stress histories in end-restrained RC ties. Three practically relevant representations of imposed deformation are compared: end-displacement-controlled axial deformation, prescribed shrinkage-type eigenstrain in concrete and thermal loading-inducing strains in concrete and reinforcement. The displacement-controlled model is validated against corresponding laboratory tests, whereas the shrinkage- and temperature-based representations are examined as comparative numerical scenarios under nominally equivalent imposed-strain levels. The results are interpreted in terms of evolving transfer lengths and tension stiffening. It is demonstrated through the analyses that matching a nominal imposed-strain level does not ensure mechanical equivalence between the three loading representations considered. Although the three approaches may produce comparable final crack widths in some of the analysed cases, they result in markedly different reinforcement-stress histories, transfer-length evolution and steel–concrete force redistribution. This distinction is important when the crack-width analysis is coupled with reinforcement-stress assessment or when imposed strains are superposed with mechanical loading.

## 1. Introduction

Imposed strains are a primary type of loading in semi-massive and massive RC structures. Their variation induces tensile stresses and early-age cracking, often excessive. Cracking induced by imposed strains seldom reaches a fully stabilised state; however, primary consideration is given to the final stabilised cracking stage in the provisions of EN 1992-3:2006 [1] and EN 1992-1-1:2023 [2]. As a result, a crack spacing smaller than that observed in practice may be assumed in code-based procedures, while the cracking history and its influence on crack widths may be neglected. In the initial cracking phase, the slip region on both sides of a crack may extend over the full transfer length, which can contribute to discrepancies when only a few widely spaced cracks are observed in laboratory tests or real structures [3]. From a design perspective, the critical issue is not only the final crack spacing but also the early cracking stage, during which transfer lengths may extend over large parts of the member and govern peak reinforcement stresses and crack widths.

Under end restraint, the formation of a crack reduces the axial restraint force and causes a decrease in concrete tensile stresses throughout the member. This decrease is greatest in the immediate vicinity of the newly formed crack, while the smaller stress reductions occur in the remaining parts of the member. Bond transfers tensile force from the reinforcement back into the concrete away from the crack, but another crack can form only when the concrete tensile strength is reached again outside the stress-relieved region. Further imposed deformation is therefore required to rebuild tensile stresses, while existing cracks reopen and widen. During this non-stabilised stage, cracks may remain widely spaced, and their widths develop through the accumulation of relative steel–concrete displacement over the transfer regions on both sides [3]. Consequently, assuming stabilised crack spacing prematurely can underestimate the length contributing to crack opening and hence the maximum crack width. Formation of a subsequent crack causes renewed force relaxation and may temporarily reduce the widths of existing cracks.

The crack-width and crack-spacing prediction in reinforced concrete has recently been revisited from several complementary perspectives. Schlicke et al. [3] emphasised that maximum crack-width calculation should distinguish between different cracking phases and should explicitly account for bond behaviour and transfer length. Sturm et al. [4] proposed a hybrid deterministic–probabilistic approach in which the crack spacing and crack width are related to reinforcement–concrete interaction using partial-interaction mechanics. More broadly, van der Esch et al. [5] showed that numerous crack-width and crack-spacing formulae have been proposed in the literature, differing substantially in their assumptions, field of application and mechanical background. It is confirmed by the findings of these studies that crack-width prediction remains an active research topic and that the assumed crack-spacing model and bond-transfer mechanism strongly affect calculated crack widths.

For members subjected to imposed strains, the problem is even more specific because cracking is governed not only by direct loading but also by restraint, shrinkage or temperature-induced deformation. Elwakeel et al. [6] analysed cracking performance in edge-restrained RC walls and showed the relevance of restraint degree, imposed deformation and reinforcement arrangement for crack development. Jędrzejewska et al. [7] reviewed standardised models for crack-width control due to restraint of imposed strains, while Jędrzejewska et al. [8] demonstrated that standardised predictions for edge-restrained members may differ significantly depending on the adopted cracking stage, material parameters and restraint conditions. It was further confirmed in recent experiments by El Khoury et al. [9] that cracking in edge-restrained wall-type members remains difficult to predict accurately using simplified design models.

In parallel, the importance of local force transfer between reinforcement and concrete has been further highlighted through recent bond-oriented modelling. Galkovski et al. [10] proposed a stress-field model for bond in reinforced concrete ties, showing that bond cannot be reduced to a purely empirical parameter when local cracking mechanisms and force transfer are analysed. Nevertheless, primary attention is still given to the final crack pattern, calibrated crack-spacing expressions or global crack-width prediction in most existing models and design procedures. Less attention has been devoted to the stepwise evolution of cracking under imposed strains, including the redistribution of internal forces, the shortening of transfer lengths and the resulting reinforcement-stress histories. Although the individual imposed-strain representations have been investigated in previous studies, to the author’s knowledge, the three approaches considered here have not been directly compared within a common numerical framework using consistent material, bond–slip and discretisation assumptions. In the present study, this gap is addressed by examining how the choice of imposed-strain representation affects crack widths, reinforcement-stress histories and force-transfer mechanisms.

In more recent experimental studies, cracking and force transfer in reinforced concrete ties were further investigated. Rimkus and Gribniak [11] examined cracking and deformations in ties reinforced with multiple bars. Galkovski et al. [12] combined distributed fibre optical sensing with digital image correlation to investigate reinforcement strains, bond stresses and crack development. Sensitivity to parameters such as rib geometry and bar diameter was identified in their results, and the need to assess local force-transfer predictions in addition to crack widths was highlighted.

**Novelty and contributions**. This study quantified how similar cracking measures can coexist with different reinforcement-stress histories in displacement-controlled, shrinkage-type and cooling representations. Although these actions define different physical problems, their consequences are compared within a common bond–slip FE framework using consistent geometry, material laws and discretisation. The contribution comprises: (i) comparison of crack spacing, widths and reinforcement stresses; (ii) interpretation of the differences through evolving transfer lengths and internal force redistribution; and (iii) experimental assessment of Model 1 and comparison with code-based crack-width procedures. One concrete mix and two bar diameters are considered in the main comparison; the Model 1 crack-width assessment is extended to additional concrete and reinforcement configurations in Section 7.4.

## 2. Engineering Methods of Imposed Strains Modelling

The use of thermal analogies to represent concrete shrinkage is illustrated in published FE applications. Vargas et al. [13] applied an equivalent temperature reduction to the walls and slabs of a building. Grosel and Pakos [14] simulated shrinkage by cooling the concrete while excluding thermal deformation of the reinforcement. These examples demonstrated the practical use of alternative strain-imposition procedures, but did not establish general interchangeability of displacement-controlled loading, concrete shrinkage and physical cooling.

A further motivation follows from Equation (9.11) of EN 1992-1-1:2023 [2], which uses the same mean steel–concrete strain-difference expression for direct loading in the stabilised cracking stage and imposed strains under dominant end restraint in the crack-formation stage. This common expression motivates comparison of the loading situations.

**Figure 1:**
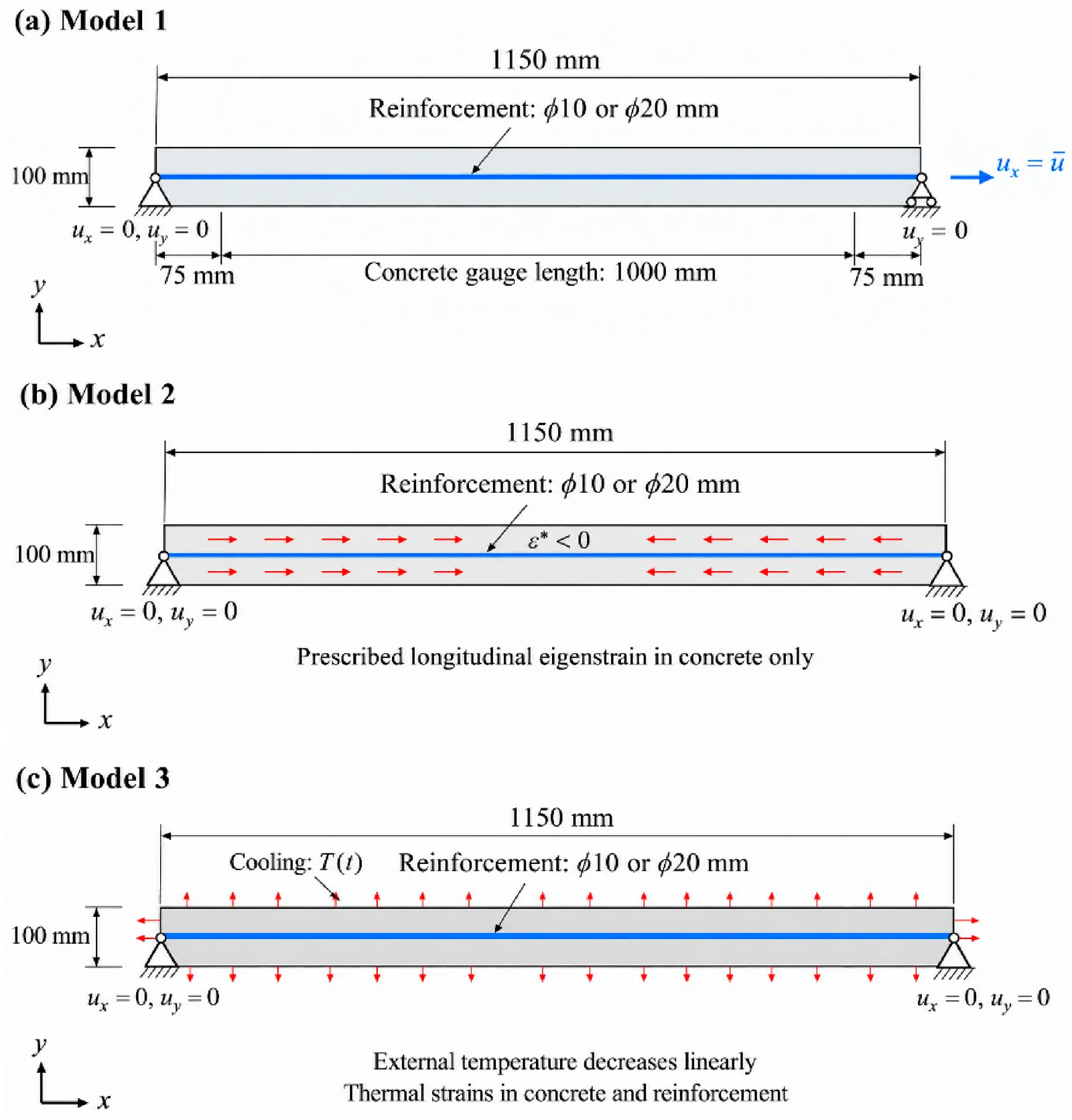
Geometry, reinforcement-end restraints and loading representations: (**a**) prescribed reinforcement-end displacement, with strain evaluated over the 1000 mm concrete gauge length; (**b**) longitudinal free contraction prescribed in concrete only; and (**c**) cooling-inducing free thermal strains in concrete and reinforcement. Actual deformations depend on restraint and steel–concrete interaction. Red arrows indicate contraction tendency in (**b**) and heat loss in (**c**).

The three loading representations, denoted M1, M2 and M3, are illustrated in Figure 1. Model 1 prescribes an axial displacement at one reinforcement end, while fixing the opposite end, with no eigenstrain; it corresponds to the reference experiments (Figure 1a). Model 2 prescribes longitudinal contraction of concrete only (Figure 1b), whereas the Model 3 induces thermal contraction of both concrete and reinforcement (Figure 1c). Both maintain fixed reinforcement-end separation. Models 2 and 3 therefore represent distinct shrinkage-type and cooling problems.

Here, “nominally equivalent imposed strain” denotes matching numerical magnitudes of the model-specific strain measures used for comparison, rather than equivalence of the mechanical states. In Model 1, this measure is the resulting average axial strain, calculated by dividing the relative axial displacement of the concrete points bounding the central 1000 mm gauge length by that gauge length; it therefore includes crack openings.

In Model 2, it is the magnitude of the prescribed longitudinal concrete eigenstrain. In Model 3, it is the magnitude of the nominal free thermal strain of concrete, calculated as *ε_imp_* = |*α_c_*Δ*T_c_*|, where Δ*T_c_* is the change in the calculated, approximately uniform temperature of the member relative to its initial temperature. Throughout the manuscript, *ε_imp_* denotes the model-specific comparison strain defined above; it does not represent the same physical strain quantity in all three models. Thus, the comparison does not impose equal total elongations, equal average mechanical strains or equal restraint forces across the three models. The resulting reinforcement stresses and internal force distributions are outputs of the respective boundary-value problems. Three levels of equivalence are distinguished. Physical equivalence requires representations of the same loading and restraint problem, including consistent free strains in concrete and reinforcement, or a demonstrated transformation that preserves the governing problem. Strain-level equivalence denotes only equality of the numerical values of the comparison measures defined above. In particular, the resulting gauge strain in Model 1 and the free concrete strains in Models 2 and 3 are different physical quantities. Response equivalence concerns agreement in specified outputs, such as crack widths or reinforcement stresses, over a defined loading range and within an explicitly stated tolerance. Agreement in one output at one stage does not establish agreement in other outputs or throughout the loading history. In the present comparison, the strain-level matching is established and differences in response are examined; physical equivalence is not assumed.

## 3. Reference Experiments and Validation Strategy

Model 1 is assessed against the displacement-controlled tests of Farra and Jaccoud [15], selected for their matching loading arrangement and detailed stage-by-stage documentation of crack development. This choice reflects data relevance and completeness rather than the absence of recent studies. The crack-width assessment is extended in Section 7.4 to the 4 × 10-C40-L specimens tested by Gribniak et al. [16] in 2020.

Models 2 and 3 are comparative numerical scenarios using the same geometry, material laws and discretisation. Their differences from Model 1 quantify the effects of loading and restraint conditions, not experimental prediction errors. Validation requires tests under corresponding shrinkage or cooling conditions; Model 1 is not assumed to be generally preferable for those problems.

Given the large number of results presented later in the paper, six specimens from Farra and Jaccoud [15] were selected as reference elements. These include elements marked with symbols: N10-10-1, N10-10-2, N10-10-3, N10-20-1, N10-20-2, N10-20-3. N10 denotes the concrete mix with 275 kg/m^3^ of Type N cement (i.e., without additives). This concrete was treated by Farra and Jaccoud [15] as the reference mix and was adopted for the main numerical comparison. The next number in the specimen description (10 or 20) indicates the rebar diameter, while the last digit denotes the specimen number within a given series. In the figure legends, N10-1, N10-2 and N10-3 are abbreviated labels for specimens N10-10-1, N10-10-2 and N10-10-3, respectively. Similarly, N20-1, N20-2 and N20-3 denote specimens N10-20-1, N10-20-2 and N10-20-3. The cross-section was 0.10 m × 0.10 m and the length was 1.15 m. The elements were reinforced centrally with a single ϕ10 or ϕ20. The reinforcement ratios of specimens N10-10 and N10-20 were 0.78% and 3.14%, respectively. These two configurations allow comparison of contrasting reinforcement ratios within the adopted geometry, but do not constitute a systematic investigation of reinforcement ratio, bar diameter or concrete cover.

## 4. Bond–Slip Behaviour and Crack Formation in RC Members

In RC members, the formation and spacing of cracks are governed by the transfer of tensile forces from the rebar to the surrounding concrete through bond. For deformed bars, bond results from adhesion at the steel–concrete interface, friction mobilised under radial pressure and mechanical interlock due to the rib geometry. It has been shown experimentally that the contact force, acting in front of the ribs, is inclined relative to the bar axis, which generates both longitudinal shear stresses (bond) and radial stresses that may lead to splitting of the concrete cover [17,18,19,20]. The concrete around the bar therefore behaves as a tension ring.

This bond mechanism is commonly represented by a local bond stress–slip law with an ascending branch, a peak bond stress and, for larger slips, a descending branch associated with concrete crushing and splitting of the cover [20,21]. Crack formation and serviceability crack widths in ordinary RC members are associated with relatively small slips, usually not exceeding a few tenths of a millimetre. In this range, the response is governed primarily by the ascending branch of the bond law, so its accuracy is crucial for predicting crack initiation, crack spacing and tension stiffening.

In the present study, crack formation and crack widths are described by the mechanics-based model of König and Tue [22]. In this formulation, the local bond stress at the steel–concrete interface is related to the local slip by a power law, *τ*(*s*) = *C*·*s^α^*, where *s* denotes the relative longitudinal displacement between steel and concrete. The parameter *C* depends mainly on concrete compressive strength and bar surface characteristics, whereas *α* reflects bond quality. For ribbed bars under serviceability conditions, König and Tue [22] recommend *C* = 0.31·*f*_*c*,*cube*_ and *α* = 0.3. The mean cube compressive strength was estimated as *f*_*c*,*cube*_ = 36.9 MPa, consistent with the cylinder-to-cube strength ratio of 0.81 reported by Pacheco et al. [23] (29.9/0.81 ≈ 36.9 MPa), giving *C* = 11.44 for stresses in MPa and slip in mm. The same local bond–slip relationship was used for both ϕ10 and ϕ20 reinforcement. Bar diameter and reinforcement ratio entered the analyses through the reinforcement geometry, including its cross-sectional area and bond perimeter, without separate calibration of the local bond law. Thus, differences between the two configurations reflect their response under a common constitutive assumption.

The modelling is restricted to the ascending (pre-peak) branch relevant to small slips and serviceability limit state (SLS) crack widths; therefore, post-peak bond degradation is outside the scope of the present study. In the model, an RC tie is represented as a system of steel and concrete interacting through bond, and differential equilibrium equations are derived to relate the steel-stress distribution, the local bond stress and the slip between steel and concrete. Crack formation is interpreted as localisation of slip at discrete sections where the concrete tensile strength is exceeded.

## 5. Description of Numerical Model

The numerical analyses were performed in DIANA 10.7. Specimens N10-10 and N10-20 were modelled in 2D following the geometry reported by Farra and Jaccoud [15]. Concrete was modelled using 8-node quadrilateral plane-stress elements (CQ16M), with quadratic interpolation, Gauss integration and an assigned thickness of 100 mm. Reinforcement was modelled using 3-node quadratic elements and represented by one-dimensional bond–slip rebar elements embedded in the concrete continuum. The reinforcement was modelled as linear elastic, with Young’s modulus *E_s_* = 199 GPa and Poisson’s ratio *ν_s_* = 0.30 (Table 1). No yielding or plastic deformation was included. In this formulation, the bar is connected to the surrounding concrete through zero-thickness interface elements generated automatically along the bar axis. The tangential traction–slip response of the interface was defined according to König and Tue [22]. A regular structured mesh comprised 10 elements across the specimen height and 100 along its length, with element dimensions of 10 mm transversely and 11.5 mm longitudinally. Mesh sensitivity is assessed in Section 6. Self-weight was neglected. In Model 1, the left reinforcement end was restrained in both directions, while the right end was restrained transversely and subjected to a prescribed axial displacement. In Models 2 and 3, both reinforcement ends were restrained in both directions.

Concrete in tension was modelled as linear elastic (Table 1) up to the tensile strength, followed by softening described by the Hordijk curve [24,25]. A total-strain rotating-crack model was adopted, with the crack bandwidth defined according to Govindjee et al. [26].

The final loading levels were selected to cover at least the full range of experimental strains measured over the central 1000 mm gauge length, without requiring an exact match to the final measured strain. For the ϕ10 and ϕ20 specimens, respectively, Model 1 used the final reinforcement-end displacements of 1.77 and 1.27 mm, applied in 1000 equal increments. Model 2 used final longitudinal concrete eigenstrains of −1.54‰ and −1.10‰, also applied in 1000 equal increments. Model 3 used a transient heat-transfer analysis followed by a nonlinear structural analysis. The initial temperatures were 154 °C and 110 °C, respectively, and the external temperature decreased linearly from the corresponding initial value to 0 °C over 10,000 s through a convective boundary condition on the specimen perimeter. Both analyses used 1000 time increments of 10 s. The calculated temperature fields generated thermal strains using the expansion coefficients in Table 1.

Model 3 represents idealised, approximately uniform cooling of a tie restrained at its reinforcement ends. Although the temperature field is calculated through the transient heat-transfer analysis, the adopted thermal parameters produce nearly uniform temperatures throughout the member at each time step. Restrained thermal contraction of concrete and reinforcement is therefore examined using this model, rather than the effects of significant temperature gradients. The temperature levels were selected to generate nominal thermal strains comparable with the other models, rather than reproduce a specific environmental or fire exposure. Mechanical properties are kept independent of temperature, and temperature-induced material degradation is not considered.

All nonlinear structural analyses used the regular Newton–Raphson method, a parallel direct sparse solver and a maximum of 300 iterations per increment. Relative force and displacement convergence tolerances were both 0.01; convergence was accepted when either criterion was satisfied, with reference norms updated at each increment. The analysis was configured to terminate upon non-convergence. The heat-transfer analysis used a time-integration parameter of 1.0 and a solver tolerance of 10^−8^.

**Table 1:** Material properties and bond–slip parameters adopted in the numerical analyses.

Parameter	Value	Source
Mean compressive cylinder strength of concrete, *f_cm_*	29.9 MPa	Farra and Jaccoud [15]
Mean tensile strength of concrete, *f_ctm_*	2.13 MPa	Farra and Jaccoud [15]
Young’s modulus of concrete in tension, *E_ct_*	27.5 GPa	Farra and Jaccoud [15]
Poisson’s ratio of concrete, *ν_c_*	0.20	EN 1992-1-1:2004+A1:2014 [27]
Fracture energy, *G_f_*	0.135 N/mm	Equation (5.1-9) of Model Code 2010 [28]
Young’s modulus of steel, *E_s_*	199 GPa	Farra and Jaccoud [15]
Poisson’s ratio of steel, *ν_s_*	0.30	EN 1993-1-1:2005 [29]
Thermal expansion coefficient of concrete, *α_c_*	10 × 10^−6^ 1/K	EN 1992-1-1:2004+A1:2014 [27]
Thermal expansion coefficient of steel, *α_s_*	12 × 10^−6^ 1/K	EN 1993-1-1:2005 [29]
Bond–slip law	*τ*(*s*) = *C*·*s^α^*	König and Tue [22]
Bond–slip parameter *α*	0.30	König and Tue [22]
Bond–slip parameter *C*	0.31·*f_c_*_,*cube*_	König and Tue [22]

## 6. Mesh Sensitivity Analysis

Since the crack widths predicted by bond–slip FE models may depend on the adopted finite-element discretization, an additional mesh sensitivity study was performed. The reference discretization described in Section 5 consisted of quadrilateral concrete elements with a characteristic size of 11.5 mm × 10 mm. To assess the influence of mesh density on the numerical results, two additional discretizations were analysed: a refined mesh with the element size reduced approximately twofold (5.7 mm × 5 mm) and a coarse mesh with the element size increased approximately twofold (23 mm × 20 mm).

**Figure 2:**
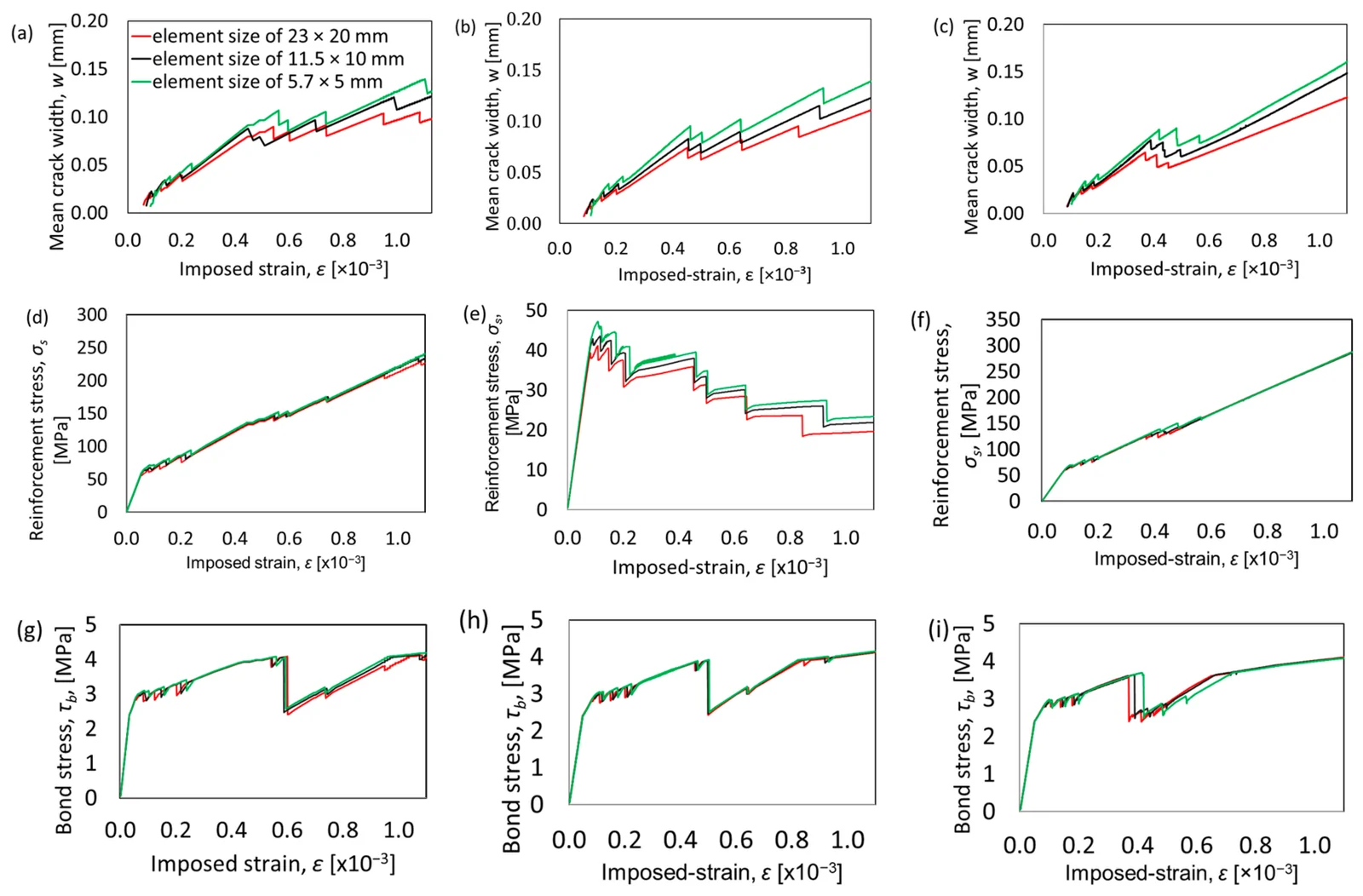
Influence of mesh density: on the predicted mean crack-width evolution for (**a**) Model 1; (**b**) Model 2; (**c**) Model 3; on the predicted reinforcement stress at the end of rebar for (**d**) Model 1; (**e**) Model 2; (**f**) Model 3; and on the predicted bond stress at the end of rebar for (**g**) Model 1; (**h**) Model 2; (**i**) Model 3.

The mesh sensitivity study covered all three imposed-strain models for the specimen reinforced with a ϕ20 mm bar. The evolution of mean crack width (a–c), reinforcement stress at the bar end (d–f), and bond stress at the bar end (g–i) is compared for the different mesh densities in Figure 2. The corresponding values at selected imposed-strain levels and percentage differences relative to the reference mesh are reported in Table 2, Table 3 and Table 4. The mean crack width was calculated as the average width of all active surface cracks identified at each analysed level.

The mesh sensitivity varied with the model and response quantity. At the tabulated strain levels, the coarse mesh predicted smaller mean crack widths, with differences ranging from −5.6% to −19.2% across the three models. The fine mesh yielded larger values: +2.3% to +4.3% for Model 1, +8.3% to +13.4% for Model 2, and +8.1% to +21.7% for Model 3. Reinforcement stresses were less sensitive, with absolute differences not exceeding 2.9%, 10.5%, and 4.1% for Models 1, 2, and 3, respectively.

Bond-stress differences were generally small, but pronounced deviations occurred for the coarse mesh in Model 1 (+61.2% at *ε_imp_* = 0.60‰) and the fine mesh in Model 3 (+39.2% at *ε_imp_* = 0.40‰). These isolated differences arise because cracking occurs at slightly different imposed-strain levels for the different meshes. Consequently, comparison at the same imposed strain may capture the local bond stress before crack formation for one mesh and after the associated stress redistribution for another.

The reference mesh was retained as a practical compromise between computational cost and resolution, rather than as a fully mesh-converged discretisation. The remaining sensitivity should be considered when interpreting the subsequent quantitative comparisons.

## 7. Results of Numerical Analyses

### 7.1. Element Reinforced with ϕ10 Rebar

#### 7.1.1. Cracking Process (Model 1)

**Figure 3:**
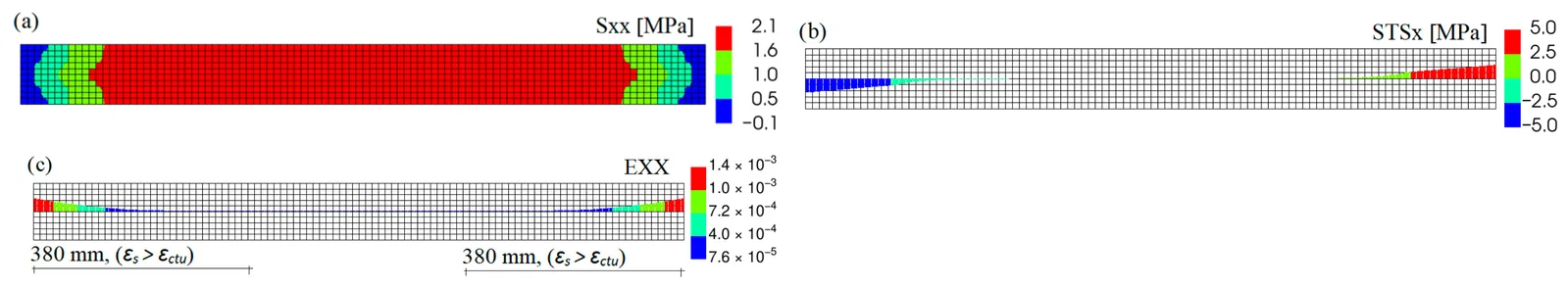
Numerical results before the first crack: (**a**) concrete Cauchy total stresses [SXX], (**b**) reinforcement interface total tractions; and (**c**) reinforcement total strains.

Immediately before the formation of the first crack, high tensile stresses develop over most of the member length (Figure 3a), whereas near the ends the tensile force in the reinforcement is gradually transferred to the concrete through bond (Figure 3b). The force is fully transferred only at the end of the transfer length; consequently, the maximum concrete tension occurs in the central part of the member. The transfer lengths shown in Figure 3c correspond to regions of strain incompatibility between concrete and reinforcement and govern the early crack-width development.

**Figure 4:**
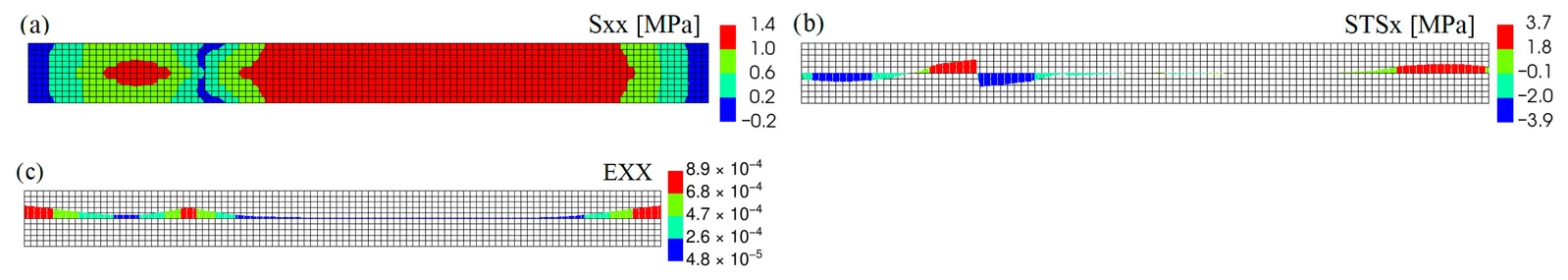
Stress and strain redistribution after the first crack: (**a**) concrete Cauchy total stresses [SXX], (**b**) reinforcement interface total tractions; and (**c**) reinforcement total strains.

Immediately after the formation of the first crack, two zones of elevated tensile stress develop on both sides of the crack (Figure 4a). The redistribution of restrained strains to the newly formed crack leads to a relaxation of tensile stresses in both concrete and reinforcement, accompanied by a reduction in bond stresses (Figure 4b). This effect becomes more pronounced for the lower reinforcement ratios. The shortening of transfer lengths caused by cracking is also illustrated in Figure 4b. The reinforcement strain distribution is shown in Figure 4c. Regions of strain compatibility between reinforcement and concrete can still be identified, indicating potential locations of further cracking.

**Figure 5:**
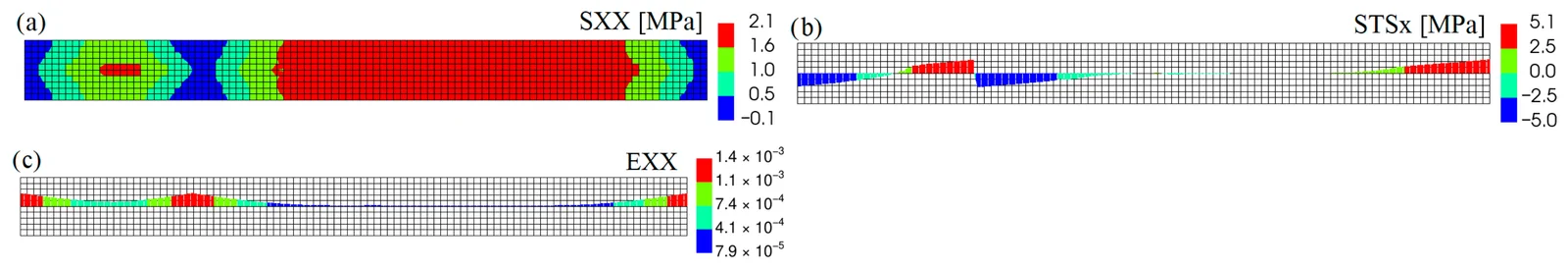
Stress and strain build-up before the second crack: (**a**) concrete Cauchy total stresses [SXX], (**b**) reinforcement interface total tractions; and (**c**) reinforcement total strains.

After the first cracking event, tensile stresses continue to build up at some distance from the crack owing to bond transfer. Increasing imposed strain leads to progressive growth of tensile stresses, bond stresses and reinforcement strains, accompanied by widening of the existing crack (Figure 5).

**Figure 6:**
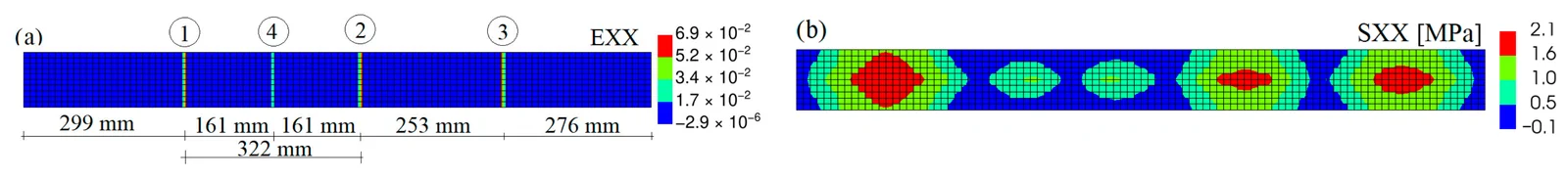
Crack pattern and stress distribution at the final imposed-strain stage for the ϕ10-reinforced tie: (**a**) concrete total strains [EXX] and (**b**) concrete Cauchy total stresses [SXX] (Model 1, *ε_imp_* = 1.4‰). The circled numbers indicate the order in which the cracks formed.

At a slightly higher imposed strain, crack No. 2 forms (Figure 6a). At the moment of cracking, similar to the first cracking described above, the imposed strains are redistributed to the newly formed crack, which is manifested by the relaxation of the entire element and the formation of new zones at higher risk of further cracking.

The cracking process repeats cyclically: bond stresses generate tensile stresses in concrete, leading to the formation of subsequent cracks at some distance from the existing ones. In the proposed model, bond stresses depend directly on local slip and therefore differ from simplified constant values assumed in code approaches.

#### 7.1.2. Crack Number and Width Evolution at the Final Imposed-Strain Stage

Finally, in the first numerical model (i.e., displacement-controlled loading applied at one reinforcement end) for the element reinforced with a ϕ10 rebar, four cracks were obtained. Their locations are shown on the map of concrete total strains EXX (Figure 6a), and the sequence of their formation is numbered 1 to 4. Between widely spaced cracks, the concrete relaxation zones extend over larger parts of the member, reflecting longer transfer lengths in the early cracking stage. The stress distribution indicates that further cracking may still occur between the left-hand end of the member and crack No. 1.

The obtained strain distributions are consistent with the cracking phases described by Schlicke et al. [3]. In the early stage, only a few widely spaced cracks are present and large parts of the member remain in strain compatibility. With increasing imposed strain, the transfer lengths shorten and the difference between reinforcement and concrete strains increases progressively. Finally, in the stabilised cracking stage, reinforcement strains exceed concrete strains along the entire member length and further loading leads mainly to the widening of existing cracks.

**Figure 7:**
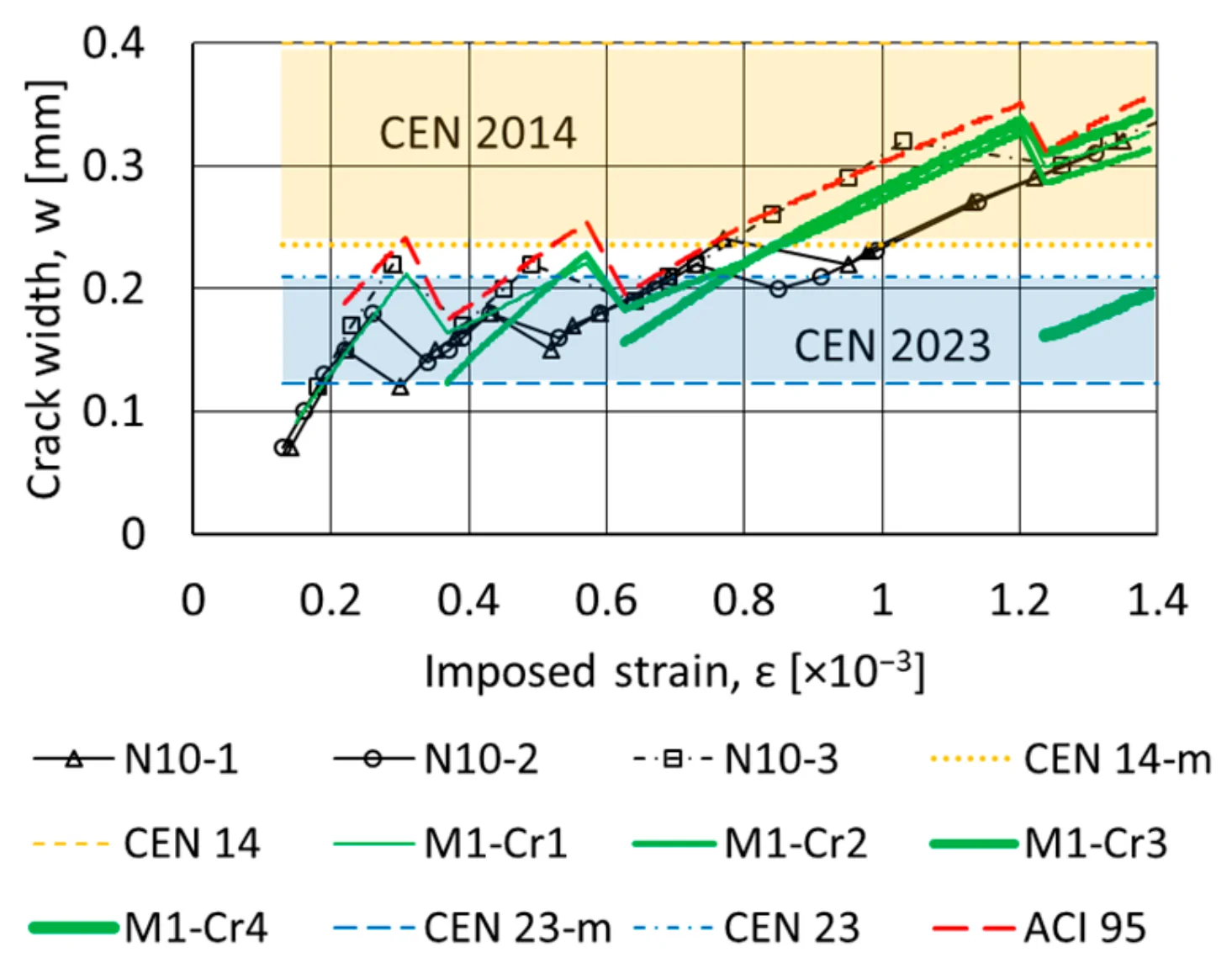
Crack widths as a function of imposed strain (Model 1, rebar ϕ10 mm).

At the first cracking stage, i.e., up to the imposed-strain level of 0.64‰, widely spaced cracks (Numbers 1–3) form. Next, crack No. 4 is formed only at the strain of 1.25‰ (Figure 7). In the figure legends, Cr1, Cr2, etc., denote individual numerical cracks numbered in their order of formation within each model. A combined label such as M2-Cr1 denotes crack No. 1 in Model 2. In reinforcement-stress plots, the same label identifies the stress evaluated at the corresponding crack section, whereas “rebar end” denotes the stress at the reinforcement end. The changes in the width of these cracks for particular values of imposed strains were very similar to the results of laboratory tests. Crack widths were measured on the element surface, consistent with the crack-width evaluation assumptions in EN 1992-1-1:2004+A1:2014 [27] and EN 1992-1-1:2023 [2]. Crack widths decreased immediately after each cracking event and increased with the increase in strains.

In Figure 7, the calculated crack-width evolution is compared with the laboratory measurements reported by Farra and Jaccoud [15], represented by black lines, and with the predictions according to EN 1992-1-1:2004+A1:2014 [27], EN 1992-1-1:2023 [2] and ACI 1995 [30]. The predictions according to EN 1992-1-1:2004+A1:2014 [27] and EN 1992-1-1:2023 [2] differ significantly owing to different assumptions regarding crack spacing and the mean strain difference between reinforcement and concrete. EN 1992-1-1:2023 [2] tends to underestimate crack widths in the stabilised cracking stage, whereas EN 1992-1-1:2004+A1:2014 [27] provides conservative predictions over the entire imposed-strain range. The expression adopted in ACI 1995 [30] and originally proposed by Gergely and Lutz [31] (Equation (1)) shows good agreement with both the numerical and experimental results:

(1) $$w=0.076\cdot \sqrt[{3}]{d_{c}A}\cdot \sigma_{s}\cdot 10^{-3},$$

where *w* is the predicted maximum crack width at the concrete surface; *d_c_* is the distance from that surface to the centre of the nearest reinforcing bar; *A* is the effective tension area of concrete surrounding the reinforcement and having the same centroid as the reinforcement, divided by the number of bars; and *σ_s_* is the calculated tensile stress in the reinforcement.

#### 7.1.3. Reinforcement Stresses and Strains at the Final Stage of Imposed Strains

**Figure 8:**
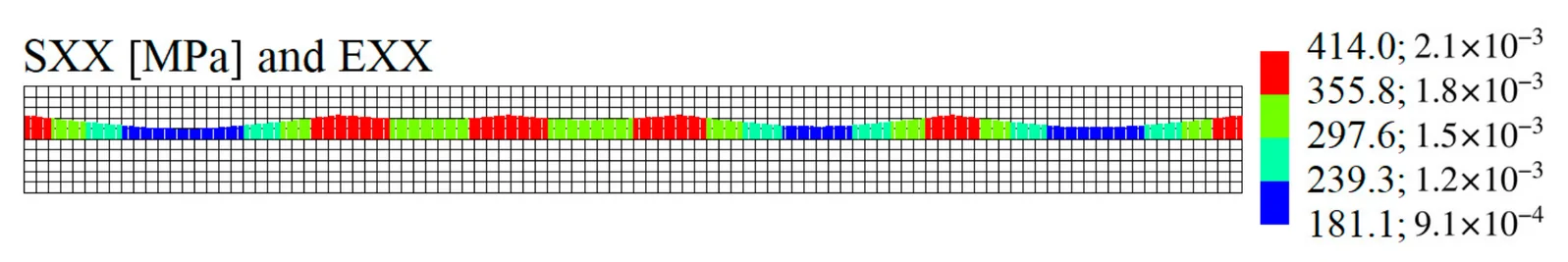
Reinforcement Cauchy total stresses and total strains (*ε_imp_* = 1.4‰) (the shared colour scale lists paired values of SXX [MPa] and EXX [–], respectively).

The stress and strain distributions in the ϕ10 reinforcement at the final imposed-strain level, *ε_imp_* = 1.4‰, are illustrated in Figure 8. The reinforcement stress (SXX) and total-strain (EXX) fields were extracted separately from DIANA. The adopted linear-elastic reinforcement model gives *σ_s_* = *E_s_*·*ε_s_*; therefore, the two fields have proportional spatial distributions. A single colour map with paired stress and strain values in the legend is presented in Figure 8. Within each pair, the reinforcement stress in MPa is listed first, followed by the corresponding dimensionless total strain.

Peak steel stresses of about 400 MPa occur at the crack sections and near the loaded bar ends. Minimum stresses of approximately 180–200 MPa occur midway between widely spaced cracks, where concrete tensile stresses are highest. Between closely spaced cracks (cracks 1, 4, 2), the steel stresses are about 320–350 MPa, indicating larger steel–concrete strain differences over these lengths.

For the analysed specimens, the tensile concrete area *A_ct_* is approximately equal to the effective tensile area *A_ceff_*; consequently, reinforcement stresses at first cracking are relatively low compared with later cracking stages.

The minimum reinforcement strain of 0.91‰ considerably exceeds the tensile strain capacity of concrete (0.077‰), indicating the presence of distributed microcracking between visible cracks. Consequently, unlike in simplified analytical models, local crack initiation cannot be identified solely from the comparison between reinforcement strain and concrete tensile strain capacity.

**Figure 9:**
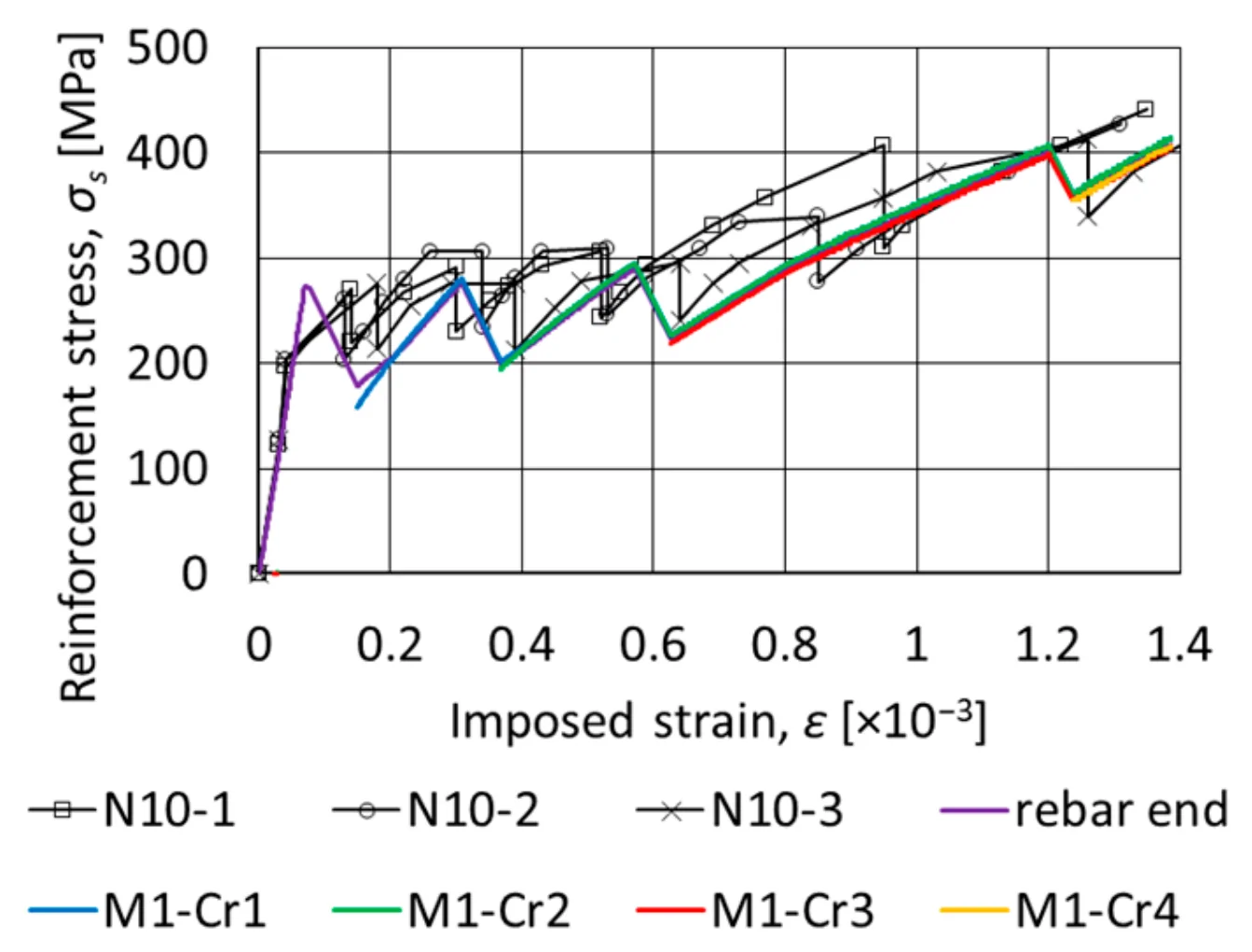
Stresses in reinforcement as a function of imposed strain (Model 1, rebar ϕ10 mm).

The evolution of reinforcement stresses at individual crack sections and at one end of the member is shown in Figure 9. Overall, the agreement with the laboratory test results is satisfactory. A certain underestimation of calculated stresses can be observed, locally reaching approximately 100 MPa depending on the cracking stage. However, the general trend of stress development and the final stress levels are consistent with the experimental results.

#### 7.1.4. Relative Displacements and Bond Stresses

**Figure 10:**
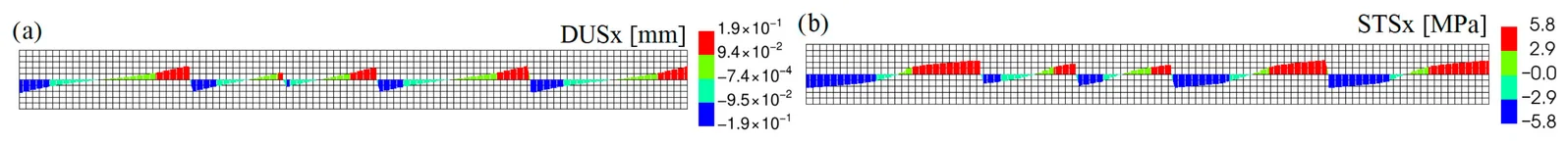
Bond–slip response at the final imposed-strain stage for the ϕ10-reinforced tie: (**a**) reinforcement relative displacements and (**b**) reinforcement interface total tractions (Model 1, *ε_imp_* = 1.4‰).

The relative interface displacements along the rebar are shown in Figure 10a. The extreme values occur at the crack faces and are higher when the spacing between adjacent cracks is larger. Their variation along the rebar length is approximately linear. The corresponding interface tractions at the final strain level are shown in Figure 10b. The smaller the relative rebar displacement, the lower the bond stress.

#### 7.1.5. Results of Analyses from Model 2

The different pre-cracking stress states can be interpreted using elastic strain decomposition and axial equilibrium. Analytical treatments of restrained thermal loading and shrinkage distinguish these imposed-deformation mechanisms [32], while bond–slip formulations account for local force transfer between the steel and concrete [33]. In the following one-dimensional derivation, an initially stress-free member, linear-elastic materials before cracking, constant material properties and no creep are assumed; tension and extension are taken as positive. The constituent stresses are

(2) $$\sigma_{c}=E_{c}(\varepsilon_{c}-\varepsilon_{c}^{*}),\sigma_{s}=E_{s}(\varepsilon_{s}-\varepsilon_{s}^{*}).$$

Here, *ε*_c_^*^ and *ε*_s_^*^ are the stress-free eigenstrains. In an uncracked interior region, where the slip gradient is small, *ε*_c_ ≈ *ε*_s_ = ε may be adopted locally. With *N* denoting the total axial force resulting from the end reactions, and *A*_c_ and *A*_s_ denoting the concrete and reinforcement areas respectively, equilibrium gives

(3) $$N=E_{c}A_{c}(\varepsilon -\varepsilon_{c}^{*})+E_{s}A_{s}(\varepsilon -\varepsilon_{s}^{*}).$$

Eliminating *ε* yields

(4) $$\sigma_{s}=\frac{E_{s}}{E_{c}A_{c}+E_{s}A_{s}}\;[N+E_{c}A_{c}(\varepsilon_{c}^{*}-\varepsilon_{s}^{*})].$$

For Model 2, *ε*_c_^*^ = *ε*_sh_ < 0 and *ε*_s_^*^ = 0. Consequently

(5) $$\sigma_{s}=\frac{E_{s}}{E_{c}A_{c}+E_{s}A_{s}}\;[N-E_{c}A_{c}|\varepsilon_{\mathrm{sh}}|].$$

Local reinforcement compression is therefore expected where *N* < *E*_c_*A*_c_|*ε*_sh_|. This is a conditional result: the sign of the steel stress depends on both the concrete eigenstrain and the restraint force. Fixing the reinforcement ends imposes zero average total steel strain over the full restrained length *L*, rather than zero strain at every section. Since *ε*_s_^*^ = 0 follows

(6) $$\frac{1}{L}\int_{0}^{L}\sigma_{s}(x)\;\mathrm{dx}=\frac{E_{s}}{L}[u_{s}(L)-u_{s}(0)]=0.$$

Thus, any local compression is balanced by tensile stresses elsewhere along the bar. Bond transfer permits this nonuniform stress distribution. The compatible-strain approximation explains the interior response but does not resolve the end transfer zones; these are represented explicitly in the bond–slip FE model.

**Figure 11:**
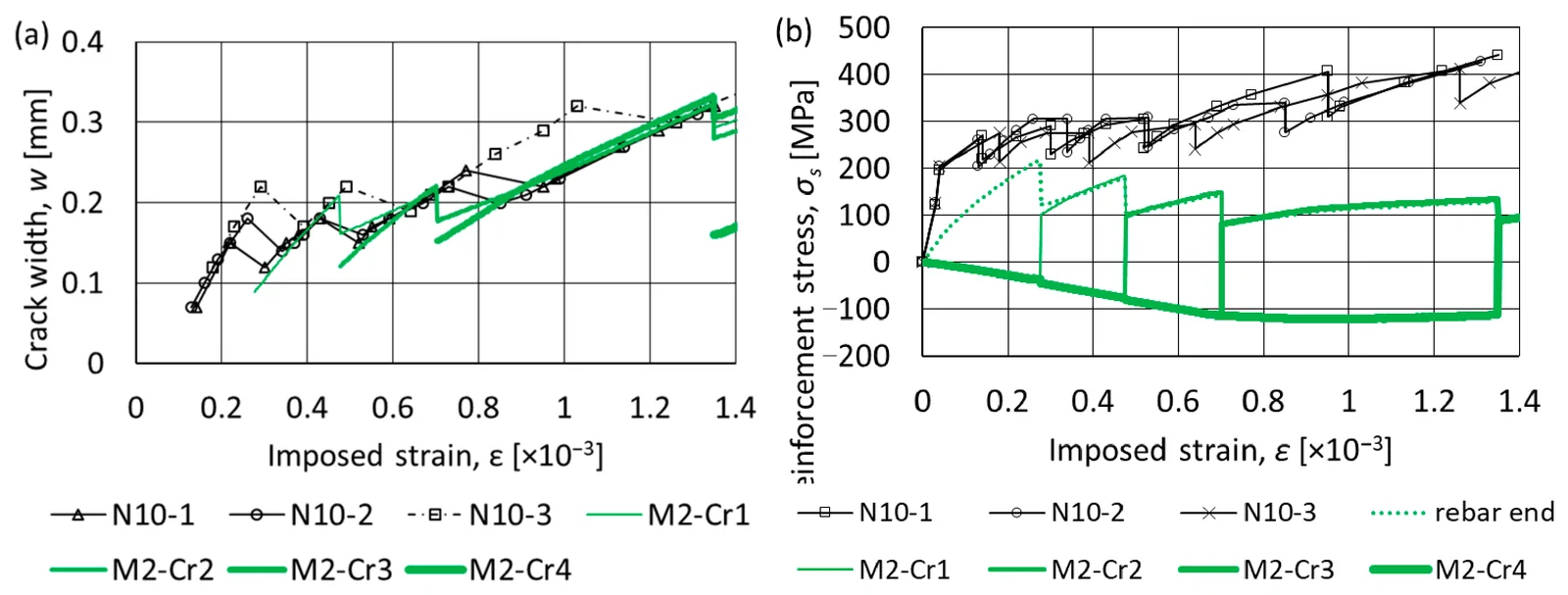
Crack-width evolution and reinforcement-stress response for the ϕ10-reinforced tie under shrinkage-type imposed strain: (**a**) crack widths and (**b**) reinforcement stresses as a function of imposed strain (Model 2).

Selected results for the second variant of imposed-strain modelling, using prescribed eigenstrain, are illustrated in Figure 11a,b. In addition, in this model four cracks were formed and the analogous distribution of stresses in concrete was found. The general tendency of crack-width changes is also analogous with the results of laboratory tests (Figure 11a). Crack widths decreased immediately after each cracking event and subsequently increased with increasing imposed strain. The agreement in the initial cracking stage is poorer than for Model 1. The cracks in the second model were formed at a later time, i.e., at slightly higher imposed strains. The maximum crack widths for certain strain levels and for the final value of imposed strain were also very close to those obtained from laboratory tests.

In uncracked sections, Model 2 generates compressive reinforcement stresses of up to 125 MPa, which is consistent with concrete shrinkage relative to reinforcement (Figure 11b). After the cracking, these stresses reverse sign and increase by approximately 250 MPa, but their final values remain below 100 MPa, considerably lower than the experimental results. Each cracking event is accompanied by temporary stress relaxation followed by renewed stress increase with increasing imposed strain.

#### 7.1.6. Results of Analyses from Model 3

For Model 3, approximately uniform cooling produces *ε*_c_^*^ = *α*_c_Δ*T* and *ε*_s_^*^ = *α*_s_Δ*T*, where Δ*T* < 0. Applying the local compatible-strain approximation, introduced in Section 7.1.5, gives

(7) $$\sigma_{s}=\frac{E_{s}}{E_{c}A_{c}+E_{s}A_{s}}\;[N+E_{c}A_{c}(\alpha_{c}-\alpha_{s})\Delta T].$$

For the adopted thermal expansion coefficients, the term (*α_c_* − *α_s_*)Δ*T* is positive during the cooling because *α_s_* > *α_c_* and Δ*T* < 0. The reinforcement tends to contract more than the concrete, and restraint of this contraction generates tensile mechanical strain. For *N* ≥ 0, the expression therefore predicts tensile reinforcement stress in the compatible interior region.

An additional result follows directly from the fixed reinforcement ends, without assuming local strain compatibility. For uniform Δ*T*, integration of *σ*_s_ = *E*_s_(*ε*_s_ − *α*_s_Δ*T*) over the full restrained bar length gives

(8) $$\frac{1}{L}\int_{0}^{L}\sigma_{s}(x)\;\mathrm{dx}=-E_{s}\alpha_{s}\Delta T>0.$$

Thus, Model 3 has a positive length-averaged reinforcement stress, whereas the corresponding average in Model 2 is zero. This global distinction does not alone determine the stress sign at every section. Locally, steel stress is governed by the mechanical strain *ε*_s_ − *α*_s_ΔT; consequently, negative total steel strain can coexist with tensile stress when *ε*_s_ > *α*_s_ΔT. These relations provide the pre-cracking mechanical interpretation, while the detailed stress distribution is governed by bond transfer, slip and the boundary conditions.

**Figure 12:**
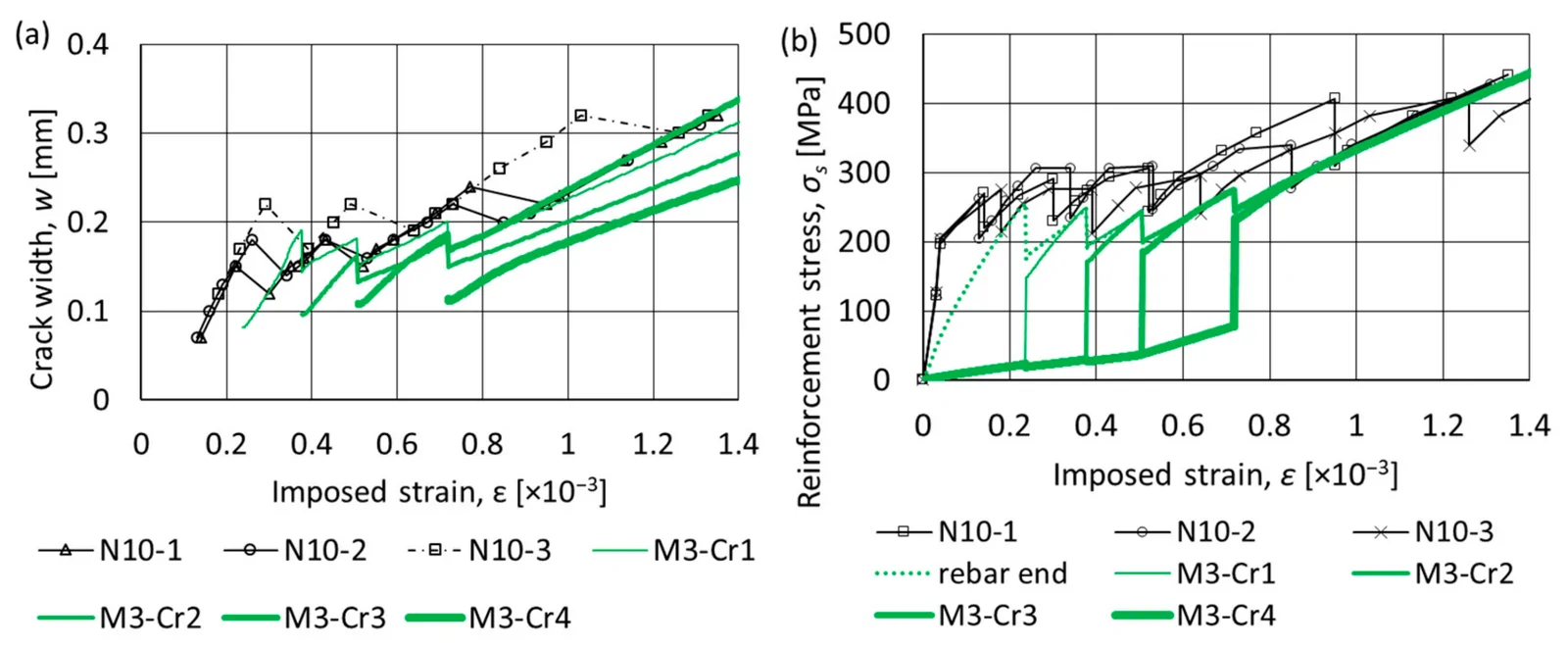
Crack-width evolution and reinforcement-stress response for the ϕ10-reinforced tie under thermal imposed strain: (**a**) crack widths and (**b**) reinforcement stresses as a function of imposed strain (Model 3).

In Figure 12a,b the results for Model 3 are presented. These results are analogous with those obtained from the two previous models as to the number of cracks and stress distribution in concrete. Up to ca. 0.8‰ of imposed strain, the width of cracks does not increase (Figure 12a), and their values are underestimated compared with the measured ones. The individual calculated crack widths should be distinguished from the experimental curves, which represent specimen-level mean crack widths. The comparison based on the mean of all calculated cracks is provided in Section 7.1.7.

Since in the model the temperature drop affects both the concrete and reinforcing steel, no compressive stresses are found in the rebar. However, negative strains can be observed in the zones most distant from the cracks. Moreover, during the crack formation the calculated stresses significantly underestimate the measured values and are in the stress range from 150 to 250 MPa (Figure 12b). After the formation of first three cracks (similar as in laboratory tests) the reinforcement stresses increase considerably. But the numerical model underestimates them compared with the measured values.

Most importantly, the final stress values along the rebar length are tensile. The strains, on the other hand (similar to Model 2), are negative in the regions between cracks that are farthest apart and positive in the cracked cross-sections and their immediate vicinity. The strain distributions indicate local imposed shortening in these zones, which results from temperature drop in the rebar, which induces tensile stresses in the rebar. In the crack region, positive strains accumulate owing to reinforcement slip relative to concrete. These results indicate that agreement in final crack width does not necessarily imply agreement in the underlying stress-transfer mechanism or reinforcement-stress history.

#### 7.1.7. Summary Comparison of Final Crack Widths

The final surface crack widths (*w*_*cr*,*1*_ to *w*_*cr*,*4*_) calculated using Models 1–3 and their arithmetic mean, *w_m,cal_*, evaluated over all calculated cracks, are summarised in Table 5. The experimental values *w*_*lab*,*1*_, *w*_*lab*,*2*_ and *w*_*lab*,*3*_ represent the mean widths of all the measured cracks in each of the three tested specimens, respectively. Their arithmetic mean is denoted by *w*_*m*,*lab*_. The comparison therefore concerns specimen-level mean crack widths, without a one-to-one correspondence between individual calculated and experimentally observed cracks. For each model, the difference at the final imposed-strain level is quantified using the signed relative difference:

(9) $$\delta_{w}\;=\;\frac{w_{m,\mathrm{cal}}\;-\;w_{m,\mathrm{lab}}}{w_{m,\mathrm{lab}}}\;\times \;100\%.$$

Negative and positive values of *δ_w_* indicate underestimation and overestimation, respectively. The same criterion is applied to all three models at the final deformation level of each reinforcement configuration. All three models underestimate the experimental mean, with the smallest difference obtained for Model 1. For Models 2 and 3, this comparison provides a reference for the numerical scenarios rather than direct experimental validation.

### 7.2. Element Reinforced with ϕ20 Rebar

#### 7.2.1. Transfer Length Between Single and Stabilised Cracking (Model 1)

**Figure 13:**
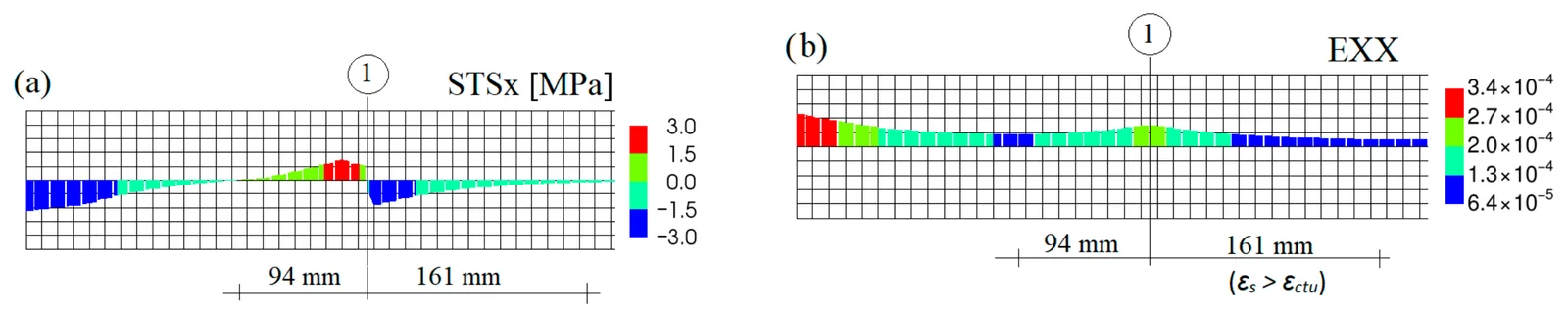
Transfer-zone response near crack No. 1 before formation of the second crack in the ϕ20-reinforced tie: (**a**) reinforcement interface total tractions and (**b**) reinforcement total strains (Model 1). The circled number 1 identifies the first crack formed.

The rebar interface total tractions in the part of the model extending from the left-hand end of the member to the region of the first crack are shown in Figure 13a. This diagram concerns the stage prior to the formation of the second crack during which an undisturbed transfer length can be observed on the right-hand side of crack No. 1. The bond stresses in this crack reach 2.29 MPa. The total rebar strain in the cross-section through the crack at this stage is 0.23‰ (Figure 13b).

**Figure 14:**
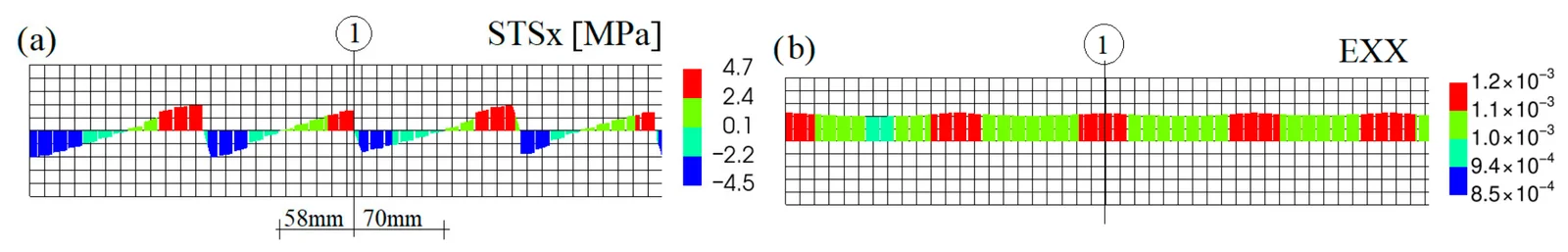
Shortening of the transfer zone near crack No. 1 before formation of the eighth crack in the ϕ20-reinforced tie: (**a**) reinforcement interface total tractions and (**b**) reinforcement total strains (Model 1). The circled number 1 identifies the first crack formed.

Comparison of these results with the bond-stress diagram for the stage just before formation of the eighth crack (Figure 14a) shows a 55% increase in the maximum bond stress, from 2.29 MPa to 3.55 MPa, accompanied by an increase in crack width. Most importantly, the transfer length on the side of the newly formed crack is considerably shortened from ca. 16 to 7 cm (i.e., by 57%). This example illustrates the evolution of transfer lengths depending on the changing crack spacing. Finally, transfer lengths equal about half the distance between adjacent cracks. The reported transfer lengths refer to the reference mesh; their mesh sensitivity was not assessed directly.

The corresponding total rebar strains are shown in Figure 14b. In the cross-section through the crack, the total rebar strains increased from 0.23 to 1.15‰, while the value of strain (at the distance of half the cracks spacing) increased from 0.13‰ to 1.04‰. These values are respectively about five and eight times higher than those observed during the single-crack stage. The reinforcement strains are several times higher than the tensile strain capacity of concrete, which further illustrates the progressive increase in strain incompatibility during crack development. This example further illustrates that, similarly to analytical models, the difference in mean strains between rebar and concrete considerably increases with the propagating cracking process.

#### 7.2.2. Results of Analyses at the Final Stage of Imposed Strains (Model 1)

**Figure 15:**
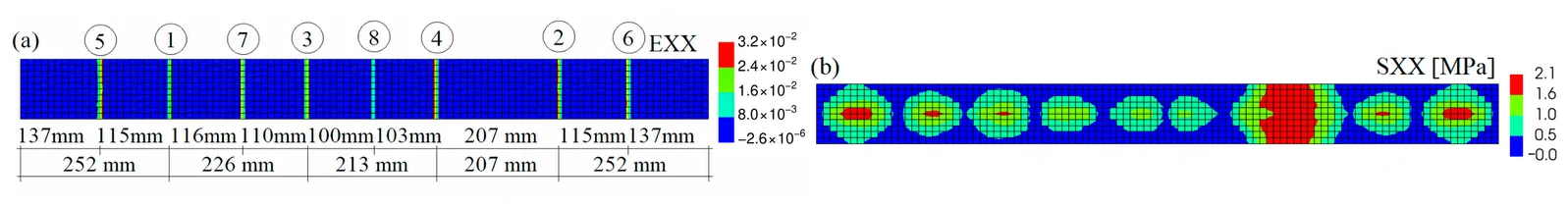
Crack pattern and stress distribution at the final imposed-strain stage for the ϕ20-reinforced tie: (**a**) concrete total strains [EXX] and (**b**) concrete Cauchy total stresses [SXX] (Model 1, *ε_imp_* = 1.1‰). The circled numbers indicate the order in which the cracks formed.

**Figure 16:**
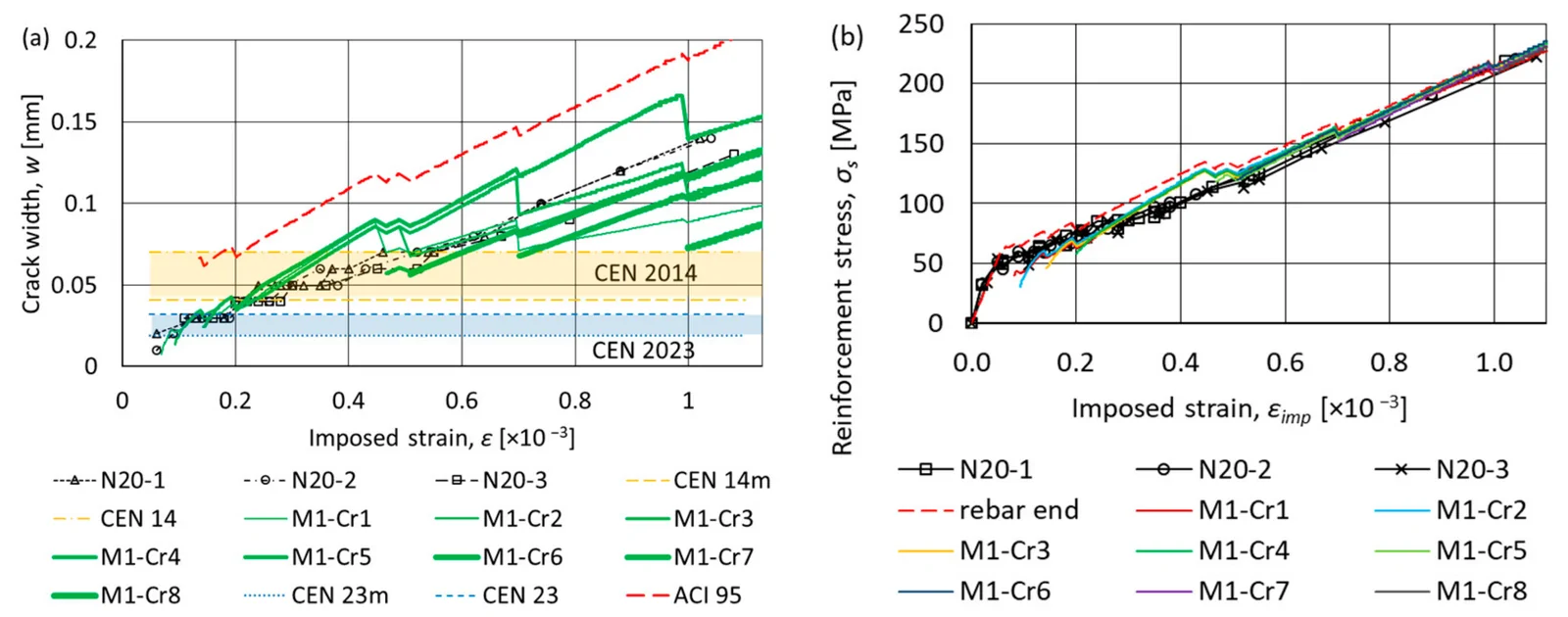
Crack-width evolution and reinforcement-stress response for the ϕ20-reinforced tie under end-displacement-controlled imposed strain: (**a**) crack widths and (**b**) reinforcement stresses as a function of imposed strain (Model 1).

**Figure 17:**
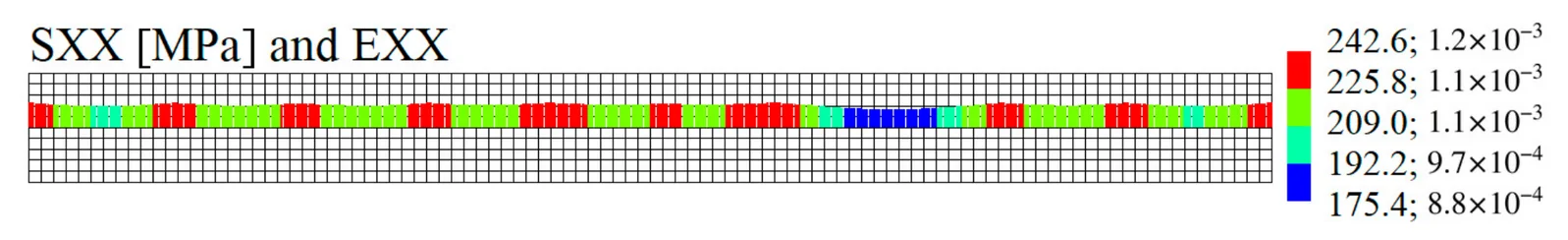
Reinforcement Cauchy total stresses and total strains (*ε_imp_* = 1.1‰).

**Figure 18:**
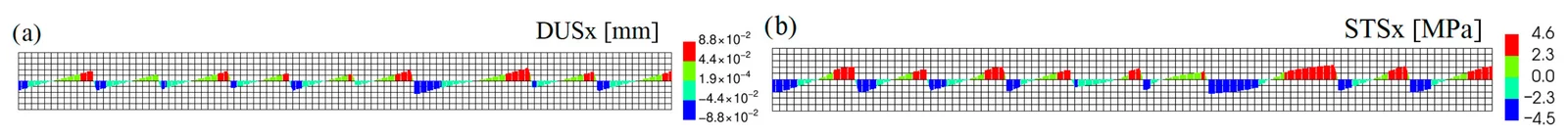
Bond–slip response at the final imposed-strain stage for the ϕ20-reinforced tie: (**a**) reinforcement relative displacements and (**b**) reinforcement interface total tractions (Model 1, *ε_imp_* = 1.1‰).

The results of numerical analysis based on Model 1 for the final stage of imposed strain (*ε_imp_* = 1.1‰) are presented in Figure 15, Figure 16, Figure 17 and Figure 18. The location of eight cracks and stress distribution in concrete are shown in Figure 15a,b, respectively. At the first cracking stage, i.e., up to the strain level of 0.20‰, cracks 1 to 4 are formed. Cracks 5 to 8, in turn, are formed only at the strain levels of 0.41, 0.51, 0.70, and 1.00‰, respectively. Good agreement in the predicted number of cracks was obtained, but individual calculated crack widths exceed the specimen-level mean measured widths in the strain range of 0.3–0.7‰ (Figure 16a), whereas some fall below these experimental means in the range of 0.7–1.1‰. The differences between the widths of particular cracks in the strain range of 0.5 to 1.1‰ are highly significant. For instance, at the final strain of 1.1‰ the crack widths ranged from 0.085 to 0.15 mm.

Practically over the entire range of imposed strains, good compatibility of stresses in the rebar was found (Figure 16b). At the final imposed-strain level of 1.1‰, the maximum reinforcement stresses at the crack sections were of the order of 234–243 MPa (Figure 17).

In Figure 16a, the shaded areas representing crack widths calculated according to EN 1992-1-1:2004+A1:2014 [27] and EN 1992-1-1:2023 [2] take a shape different from that for element types N10-10. The crack widths are estimated correctly according to EN 1992-1-1:2023 [2] to the strain level of 0.2‰, i.e., to the level of tensile force slightly exceeding the cracking force. EN 1992-1-1:2004+A1:2014 [27], however, safely predicts the results to the strain level of 0.56‰, which corresponds to exceeding the tensile force to the value of 1.3 *N_cr_*.

Compared to specimens N10-10, the underestimation according to EN 1992-1-1:2004+A1:2014 [27] becomes more pronounced at higher imposed-strain levels corresponding to larger tensile forces in the highly reinforced member. Crack widths calculated according to ACI 1995 [30] are overestimated over the entire imposed-strain range. This overestimation is 0.05 mm maximum.

The results for reinforcement stress and strain distributions (Figure 17) indicate the possibility of the formation of another crack between cracks 4 and 2. Owing to a large rebar diameter (and consequently a high reinforcement degree), rebar relative displacements are definitely smaller (Figure 18a) compared with the results obtained for an element reinforced with a ϕ10 mm rebar. It is also an effect of a greater area of rebar’s side surface, which results in lower bond stresses (Figure 18b), and narrower cracks.

#### 7.2.3. Results of Analyses Based on Models 2 and 3

**Figure 19:**
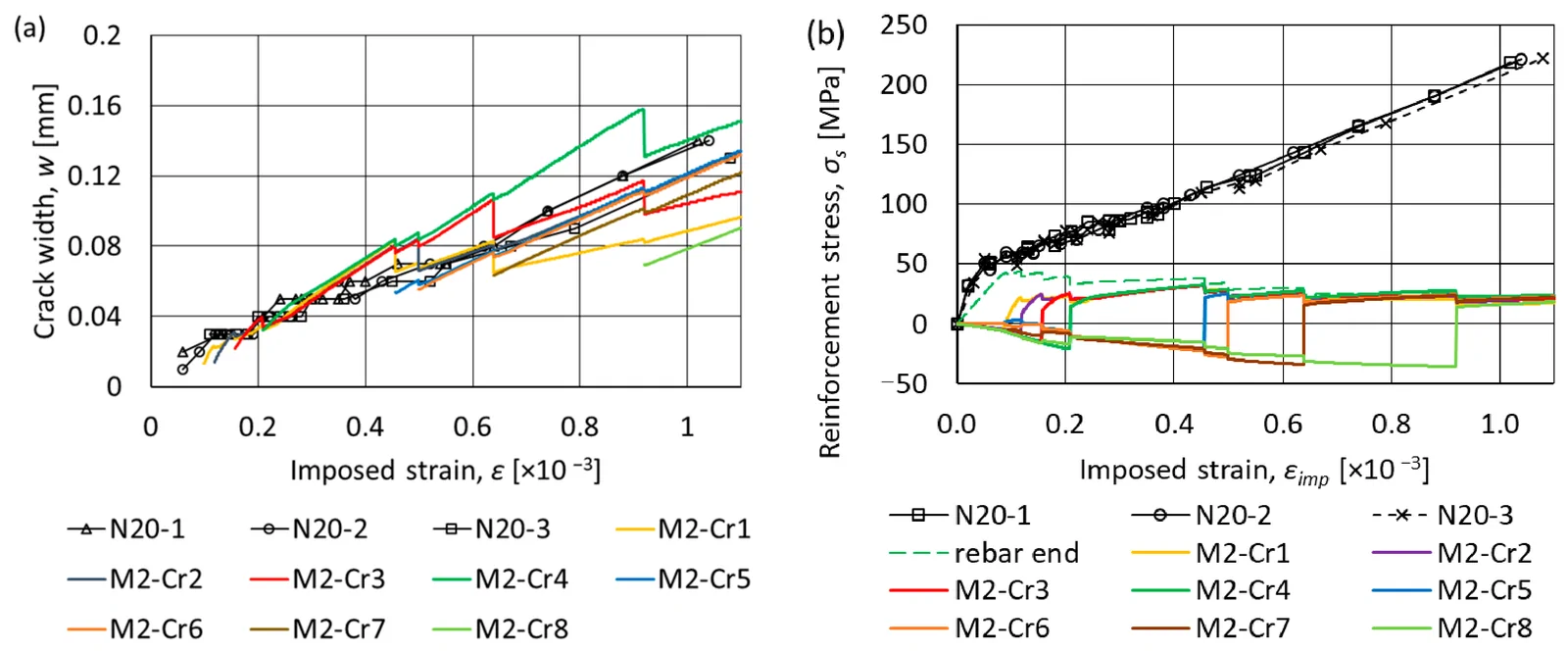
Crack-width evolution and reinforcement-stress response for the ϕ20-reinforced tie under shrinkage-type imposed strain: (**a**) crack widths and (**b**) reinforcement stresses as a function of imposed strain (Model 2).

**Figure 20:**
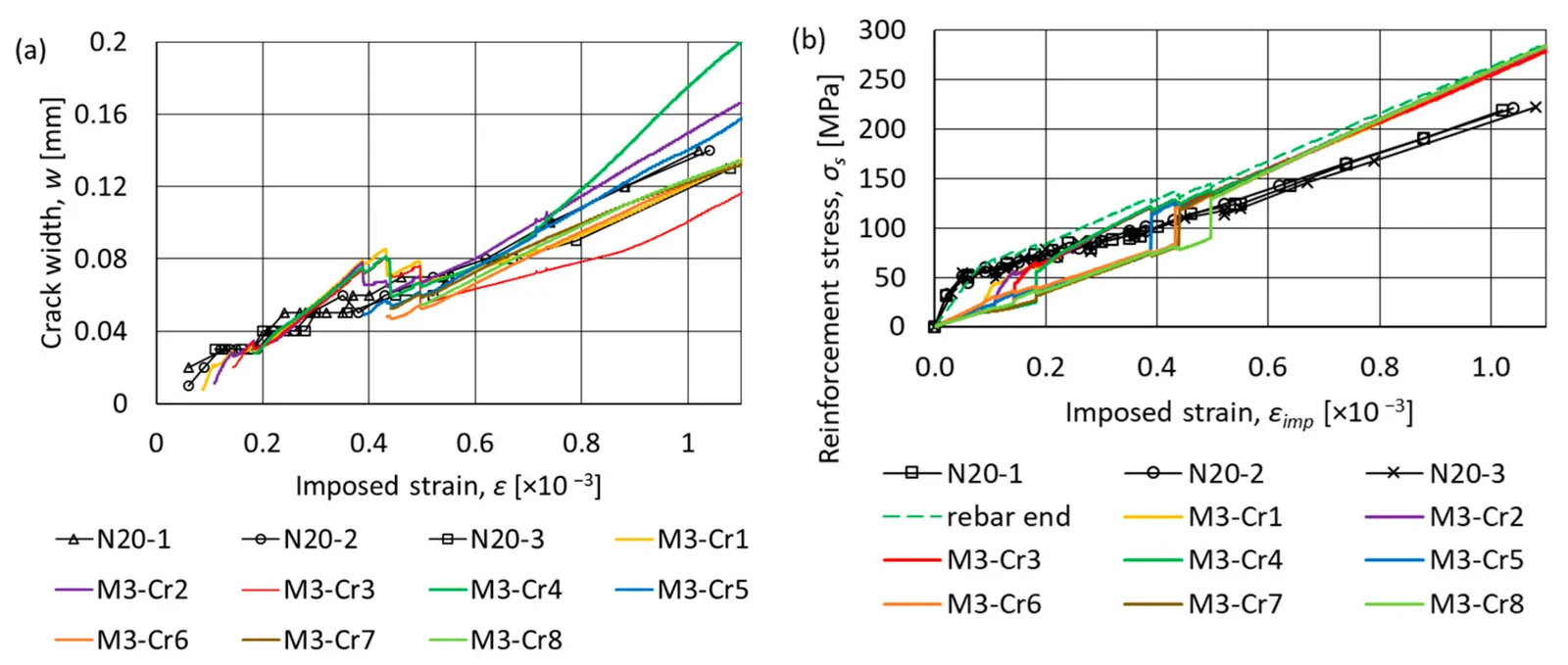
Crack-width evolution and reinforcement-stress response for the ϕ20-reinforced tie under thermal imposed strain: (**a**) crack widths and (**b**) reinforcement stresses as a function of imposed strain (Model 3).

For Model 2 (Figure 19a,b), similarly to the member reinforced with a ϕ10 mm rebar, the crack widths are comparable with the laboratory test results. In both Models 2 and 3, eight cracks developed with similar widths (Figure 19a and Figure 20a).

Models 2 and 3 produce qualitatively different reinforcement-stress histories. In Model 2 (Figure 19b), reinforcement stresses reach a mean value after cracking of ca. 25 MPa and remain at a similar level until the final load. According to Model 3, the rebar stresses are significantly overestimated in the stabilised cracking stage (Figure 20b).

The discrepancy between the similar crack-width predictions and markedly different reinforcement-stress histories confirms that the imposed-strain representation affects the mechanical path of cracking, even when the final crack pattern appears similar.

#### 7.2.4. Influence of Transfer-Length Definition on Mean Bond Stresses and Bond Coefficients (N10-20, Model 1)

Mean bond stresses were evaluated for the N10-20 specimen using Model 1 at eight selected cracking stages, corresponding to one to eight cracks. Two definitions of the integration intervals were considered to distinguish the influence of the final crack spacing from the evolution of the bond-stress distribution. The first approach uses intervals limited by the final crack spacing, whereas the second determines the transfer lengths directly from the FE bond-stress distribution at each stage.

In the first approach, the maximum interval length on each side of a crack was taken as a half the final spacing to the adjacent crack. On the outward-facing sides of the two outermost cracks, this limit was taken as a half the distance from the crack to the corresponding specimen end. If the bond stress changed sign before the prescribed limit, the interval was shortened to the first zero crossing encountered when moving away from the crack. This location was determined by linear interpolation separately at each stage. These geometrically limited intervals are used as a reference for comparison and are not interpreted as the actual transfer lengths at earlier cracking stages. The local mean bond stress was evaluated by trapezoidal integration over each interval as |*τ_bm_*| = |∫*τ*(*x*) dx|/*L*. The resulting mean bond stresses and interval lengths are presented in Table 6.

The corresponding local coefficients *k_1_* = *f_ctm_*/∣*τ_bm_*∣ are presented in Table 7. In both approaches, *τ_mean_* and *k*_*1*,*mean*_ are the unweighted arithmetic means of the local absolute mean bond stresses and local coefficients, respectively, over all crack sides present at the corresponding stage.

In the second approach, the transfer length on each side was determined directly from the FE bond-stress distribution, extending from the crack to the first zero crossing encountered on that side. The zero-crossing positions were obtained by linear interpolation independently at each stage, without imposing a limit based on the final crack spacing. The same integration procedure and definitions of the local and stage-mean quantities were used. The resulting bond stresses and transfer lengths are given in Table 8, and the corresponding coefficients are given in Table 9. Here, “transfer length” denotes the crack-side interval identified by this zero-crossing criterion.

These intervals do not necessarily cover the full specimen length. The final stage, approximately 72.44 mm at the left end and 72.48 mm at the right end, lie outside the intervals assigned to the outermost cracks. Bond stresses remain present in these end regions, but have the opposite sign to those in the adjoining crack-side intervals. They therefore contribute to end force transfer without being included in the reported crack-side means.

The comparison shows that the interval definition has a substantial influence at the early cracking stages. With one crack, the geometrically limited intervals are 57.5 and 60.4 mm long, whereas the directly identified transfer lengths are 98.0 and 378.8 mm. Using the latter reduces *τ_mean_* from 1.54 to 0.68 MPa and increases *k_1_*_,*mean*_ from 1.40 to 3.63.

The differences diminish as cracking progresses. Relative to the geometrically limited approach, the stage-specific intervals reduce *τ_mean_* by approximately 56% at the one-crack stage but only 3% at the eight-crack stage; the corresponding increases in *k_1_*_,*mean*_ are 159% and 4%, based on the rounded table values.

At the final stage, the two approaches give *τ_mean_* = 2.28 and 2.22 MPa, and *k_1_*_,*mean*_ = 0.97 and 1.01, respectively. However, the close agreement of the stage means does not imply that the zero-crossing positions coincide with the midpoints between cracks. Local differences remain: for example, at Cr4L, the mean bond stress decreases from 1.80 to 1.47 MPa, while *k_1_* increases from 1.18 to 1.45. The associated interval length increases from 51.8 to 69.4 mm. Thus, the final half-spacing remains an approximation even when its effect on the overall stage means is small.

The stage-specific results show that the inferred bond coefficient is not constant during cracking. Its mean decreases from 3.63 at the one-crack stage to 0.85 at the four-crack stage and subsequently varies between approximately 0.90 and 1.01. For comparison, EN 1992-1-1:2004+A1:2014 [27] adopts *k_1_* = 0.8, whereas EN 1992-1-1:2023 [2] adopts *k_1_* = 0.56. Expressed using the definition *k_1_* = *f_ctm_*/ǀ*τ_bm_*ǀ, the bond-stress ratios in fib Model Code 2010 [28] and fib Model Code 2020 [34] correspond to approximately *k_1_* = 1/1.8 = 0.56 for short-term instantaneous loading and for stabilised cracking under short- and long-term loading, and *k_1_* = 1/1.35 = 0.74 for long-term loading during crack formation.

For stages with four to eight cracks, the stage-specific mean coefficients are closest to the value of 0.80 adopted in EN 1992-1-1:2004+A1:2014 [27], but remain approximately 6–26% higher. At the final stage, the mean coefficient is 1.01, compared to 0.97 for the geometrically limited intervals. Agreement with standard values therefore depends on both the cracking stage and the interval definition. This comparison is limited to a 20 mm reinforcing bar and a single concrete mix and does not constitute a calibration of the standard crack-width formulations.

#### 7.2.5. Summary Comparison of the Final Crack Widths

Final mean surface crack widths for the ϕ20-reinforced tie are compared in Table 10 using the averaging procedure in Section 7.1.7 and the signed relative difference defined by Equation (9). Models 1 and 2 underestimate the experimental mean, whereas Model 3 overestimates it. Model 3 gives the smallest absolute difference for the reference mesh, but this ranking has not been established as mesh-independent (Section 6) and does not validate the thermal loading representation.

### 7.3. Axial-Force Redistribution Between Concrete and Reinforcement

**Figure 21:**
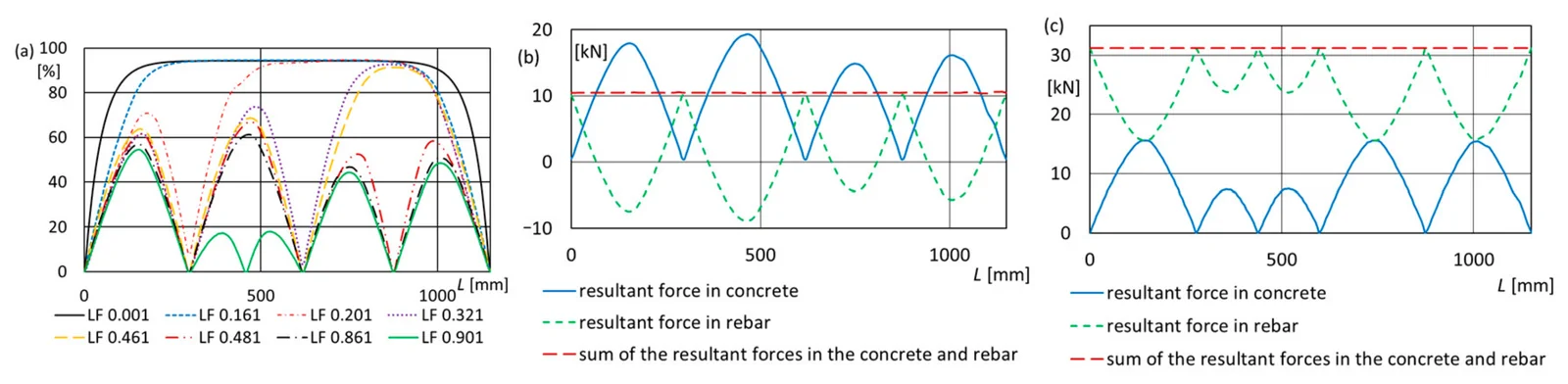
Predicted axial-force redistribution between concrete and reinforcement in the ϕ10-reinforced tie (N10-10): (**a**) imposed displacement, Model 1—percentage of the total axial tensile force carried by concrete at selected load factors; (**b**) shrinkage-type imposed strain, Model 2—axial forces in concrete and reinforcement and their sum under shrinkage-type imposed strain; and (**c**) thermal imposed strain, Model 3—axial forces in concrete and reinforcement and their sum under thermal imposed strain.

The predicted axial-force redistribution between concrete and reinforcement in the ϕ10-reinforced tie (N10-10) is presented in Figure 21 for the three imposed-strain representations: (a) Model 1, (b) Model 2 and (c) Model 3. The percentage of the total axial tensile force carried by concrete at selected load factors (LF) is shown in Figure 21a, whereas the axial forces carried by concrete and reinforcement and their sum, expressed in kN, are shown in Figure 21b,c. At the very beginning of loading (LF = 0.001), concrete carries almost the entire tensile force *N_c_*(*x*). At LF = 0.161, still before cracking but at a higher load level, the transmission zones near the ends become longer and the share of the tensile force carried by concrete is reduced. With increasing load, this contribution decreases progressively, with marked drops each time a new crack forms. The locations where the concrete contribution reaches local maxima indicate the zones in which the next crack will develop. After the formation of a few widely spaced cracks (single-crack stage), further cracking requires a substantial increase in total axial force, so that existing cracks widen significantly before new cracks appear, in line with the tension-stiffening behaviour described, for example, by Schlicke et al. [3].

For Model 2 (Figure 21b), although the global tensile force is constant, a self-equilibrated internal force component develops, consisting of a tensile force Δ*N*(*x*) in concrete and a compressive force −Δ*N*(*x*) in the rebar. Their sum is zero, but they significantly modify the local concrete stresses. In the stabilised cracking stage, the relative contribution of this self-equilibrated component increases and the amplitudes of the local “peaks” of force in steel and concrete between cracks become larger. At this stage, it is mainly this internal redistribution of force between concrete and rebar, and to a lesser extent the change in the global resultant, that governs the initiation of new cracks and their spacing; therefore, the increase in the global tensile force primarily causes the existing cracks to widen. For Model 3, the predicted axial-force distributions in concrete and reinforcement are shown in Figure 21c. Here, the thermal expansion coefficient of steel is taken 20% higher than that of concrete, so that under cooling the rebar tends to contract more than the surrounding concrete. Because the member is restrained through the rebar ends, this leads to a pronounced increase in tensile force in the steel, which is clearly visible in both cracked and uncracked sections. Cracking reduces the tensile force carried by concrete, while the total resultant remains constant along the member. Before cracking, the axial force is almost entirely carried by the concrete. When the first crack forms, *N_c_*(x) drops locally to zero and a sharp peak in *N_s_*(*x*) appears at the crack section, with a relatively long transfer length over which *N_c_*(*x*) is gradually restored. As the temperature drop increases and further cracks develop, new peaks of *N_s_*(*x*) and corresponding valleys of *N_c_*(*x*) appear at the new crack locations, reflecting the shortening of the transfer length and the redistribution of internal forces between neighbouring cracks. In the stabilised cracking stage the tensile force in the rebar is considerably higher than the “cracking force”, but the limited bond-stress capacity (especially over the shortened transmission lengths) does not allow this force to be fully transferred to the concrete. As a result, no new cracks are formed and the existing cracks mainly widen. Overall, the evolution of the steel–concrete interaction corresponds to the well-known tension-stiffening mechanism and directly governs the order of crack formation, their spacing and the widening of existing cracks.

### 7.4. Extended Validation

**Figure 22:**
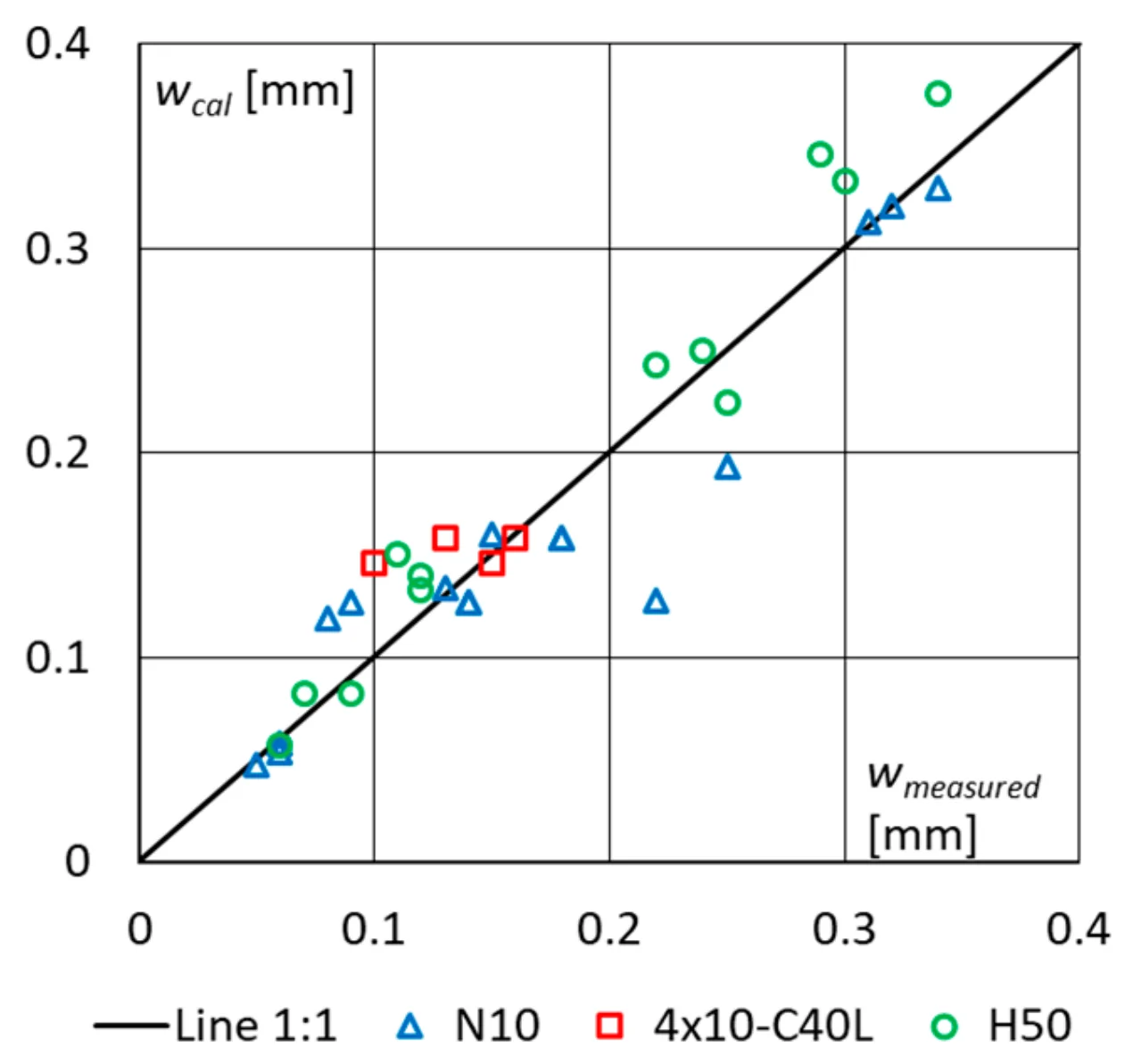
Comparison between calculated and measured crack widths for RC tie specimens.

In Figure 22, crack widths calculated exclusively using the displacement-controlled representation (Model 1) are compared with measured values for a broader set of tension members. The symbols marked “N10” correspond to specimens made with the reference concrete mix N10 from the test series by Farra and Jaccoud [15]; a subset of these tests is discussed in detail in the main part of this paper. The specimens denoted H50 originate from the same series but were cast with a significantly higher-strength concrete. In addition, two nominally identical tie specimens of the 4 × 10-C40-L configuration, with a larger cross-section and four reinforcing bars, tested by Gribniak et al. [16], were analysed.

The calculated crack widths were evaluated at the final imposed-strain level and, for the Farra and Jaccoud [15] specimens, also at an intermediate strain level. For the two nominally identical 4 × 10-C40-L specimens, crack widths were compared at two load levels for each specimen, resulting in four experimental–numerical data pairs in Figure 22. Despite the differences in concrete strength, reinforcement ratio and bar arrangement, the majority of points lie close to the 1:1 line, indicating a generally good agreement between numerical predictions and experimental measurements for all three series.

Generally good agreement between calculated and measured crack widths is observed in Figure 22 within the investigated parameter range, with slight overestimation of very small widths and underestimation of the largest widths. This assessment does not independently validate reinforcement-stress histories or local bond-stress distributions. Broader applicability requires additional experiments, particularly measurements of local reinforcement strains.

## 8. Discussion: Mechanistic Interpretation of the Numerical Results

The numerical results indicate that the cracking response of restrained RC ties cannot be interpreted solely in terms of final crack spacing or final crack width. The key mechanism is the progressive redistribution of axial force between concrete and reinforcement through bond. During the non-stabilised cracking stage, transfer lengths extend over large parts of the member, and the formation of a new crack produces local stress relaxation followed by renewed stress build-up in adjacent regions. As cracking progresses, transfer lengths shorten and the response gradually approaches the stabilised cracking stage, in which further imposed deformation mainly increases the widths of existing cracks.

A quantitative comparison of Models 1–3 for both reinforcement ratios is provided in Table 11, where the final cracking measures are presented together with spatial reinforcement-stress statistics and the maximum tensile stress attained during the preceding loading history. The results are compared at the same nominal imposed-strain level within each reinforcement configuration.

All three models predict four cracks for ϕ10 and eight for ϕ20. Relative to M1, final mean crack widths differ by −9.1% and −0.7% for M2 and M3 with ϕ10, and by +3.4% and +24.6% with ϕ20. Thus, the same final crack count and mean crack widths of the same order of magnitude are predicted using all three models, with differences in mean crack width of up to approximately 25% relative to M1.

The reinforcement stresses show substantially greater differences. For ϕ10, the final maximum tensile stresses are 414, 95 and 448 MPa for M1, M2 and M3, respectively, while the corresponding signed mean stresses are 306, 0 and 334 MPa. For ϕ20, the final maxima are 243, 23.8 and 286 MPa, and the mean stresses are 213, 0 and 263 MPa. The zero mean in M2 does not indicate an absence of reinforcement stress: local tensile and compressive stresses coexist and balance in the spatial integral. This is consistent with the fixed reinforcement-end separation and the absence of an imposed eigenstrain in the steel. In M3, the reinforcement also undergoes thermal contraction, so the same constraint on end separation requires a positive mean mechanical strain and hence a positive mean tensile stress.

The historical maxima further distinguish the responses. In M2, *σ*_*s*,*peak*_ reaches 218 MPa for ϕ10 and 43.6 MPa for ϕ20, approximately 2.3 and 1.8 times the respective final tensile maxima. These earlier peaks demonstrate that the final reinforcement-stress state does not capture the most severe tensile demand attained during loading. In contrast, the historical and final maxima coincide for M1 and M3. The final maximum absolute bond stresses range from 4.8 to 5.8 MPa for ϕ10 and from 4.1 to 4.6 MPa for ϕ20. These maxima do not, however, describe the overall shape of the bond-stress distributions.

The quantitative comparisons should be interpreted in light of the mesh sensitivity reported in Section 6. For the ϕ20 specimen, reinforcement stresses at the bar end were less sensitive than mean crack widths, whereas the largest pointwise bond-stress differences arose from shifts in the onset of individual cracking events. These checks do not establish the mesh independence of transfer lengths or local stress distributions along the entire bar, and their quantitative findings should not be extrapolated directly to the ϕ10 specimen.

The distinction between different physical mechanisms and inappropriate model substitution is essential. Different stress-transfer histories are expected for the three physical problems defined in Section 2. The additional finding is that agreement in selected cracking measures can conceal substantial differences in reinforcement demand. For example, for the ϕ20 tie, the final mean crack width in Model 2 differs from Model 1 by only +3.4%, whereas the final maximum tensile reinforcement stresses are 23.8 and 243 MPa, respectively. These are differences between numerical scenarios, rather than estimates of experimental prediction error, but they show why crack-width agreement alone cannot establish response equivalence.

Models 2 and 3, as implemented here, are inappropriate direct substitutes for the displacement-controlled Model 1 when reinforcement stresses or force redistribution are required. Matching the nominal concrete strain does not therefore preserve Model 1’s reinforcement deformation conditions and constituent free strains. Substitution requires demonstrated equivalence of the governing problem or independent verification of the required responses.

This does not make Models 2 and 3 inappropriate for their respective shrinkage and cooling problems. Nor does it exclude a restricted approximation for a particular output, provided that its accuracy is established over the relevant loading range against a physically appropriate reference and an explicit tolerance. The present results do not establish such a general approximation, and similarity in final crack widths alone is insufficient to justify it. Consequences for combined mechanical and imposed loading require separate investigation because such combinations were not analysed here.

Extension to realistic thermal exposure would require exposure-specific boundary conditions and temperature-dependent thermal, mechanical and bond properties. The thermal FE analysis of RC beams under standardised fire by Bolina and Henn [35] provides a relevant example of temperature-field modelling for such future developments.

## 9. Conclusions

Unlike the code models based on constant crack spacing, the proposed model captures the early cracking stage, in which the crack widths are governed by bar slip over the full transfer length. With increasing imposed strain, crack spacing decreases and transfer lengths shorten, which explains the predicted crack-width evolution and indicates when constant-spacing assumptions may be inaccurate.

For both analysed element types, N10-10 (ϕ10 mm) and N10-20 (ϕ20 mm), two crack-formation stages can be distinguished. In the first, new cracks form at relatively small strain increments and remain widely spaced; in the second, additional cracks develop between the earlier ones only at higher imposed strain, which shortens the transfer length.

Only Model 1 was assessed against experiments with corresponding loading conditions. Results for Models 2 and 3 remain numerical predictions requiring validation under shrinkage-type and cooling conditions; agreement with displacement-controlled crack-width measurements does not provide such validation.

The analyses of Models 1–3 indicate that the cracking behaviour of the examined RC ties is governed primarily by the local redistribution of axial force between concrete and reinforcement (tension-stiffening mechanism). This internal steel–concrete interaction controls the sequence and spacing of cracks and, in the stabilised cracking stage, leads mainly to the widening of existing cracks rather than the formation of new ones.

With the reference mesh, all three representations produced the same final crack count for each reinforcement configuration, while the final mean crack widths differed by up to approximately 25% relative to Model 1. This partial agreement in cracking measures coexisted with substantially different reinforcement-stress responses. Matching a nominal strain measure or a final crack width therefore does not establish physical or response equivalence. For the displacement-controlled reference problem, Models 2 and 3, as implemented here, cannot be justified as direct substitutes for Model 1 when reinforcement stresses and force redistribution are required.

When imposed strains exceed the cracking level, crack widths increase substantially and additional cracks may form, as confirmed by both the laboratory tests and the numerical analyses. The EN 1992-1-1:2004+A1:2014 [27] and EN 1992-1-1:2023 [2] provisions do not cover this strain range; nevertheless, EN 1992-1-1:2004+A1:2014 [27] gives a safe estimate of crack widths:

- In the entire range of strains for element type N-10-10 (ρ = 0.785%).
- To imposed-strain level of 0.56‰ for element type N10-20 (ρ = 3.14%).

The crack widths calculated according to ACI 1995 [30] show surprisingly good agreement with the laboratory results for an element of the lowest degree of reinforcement. With the increase in the reinforcement degree this agreement gets poorer, i.e., the calculated values are overestimated. This agreement is limited to the investigated specimens and strain range and does not establish the general applicability of the formula to end-restrained members.

The framework was assessed using Model 1 against fifteen axially loaded tension members. Its verified scope remains limited to the investigated configurations and pre-peak bond–slip behaviour. Broader application to bending members, larger cross-sections, other reinforcement arrangements, splitting failure or post-peak bond degradation requires further validation. The results do not establish general design recommendations for crack control or reinforcement sizing.

## Figures and Tables

**Table 2:** Influence of mesh density. Mean crack width at selected imposed-strain levels for the three models (ϕ20 mm).

Model	*ε_imp_* [‰]	Mean Crack Width Reference [mm]	Coarse Mesh Difference [%]	Fine Mesh Difference [%]
M1	0.20	0.041	−6.3	+2.3
	0.40	0.078	−9.6	+3.5
	0.60	0.083	−5.6	+3.7
	0.80	0.097	−10.3	+3.8
	1.13	0.122	−19.2	+4.3
M2	0.20	0.0365	−8.2	+8.3
	0.40	0.0717	−9.6	+12.8
	0.60	0.0840	−9.9	+13.0
	0.80	0.0995	−9.7	+13.2
	1.10	0.1215	−9.5	+13.4
M3	0.20	0.0323	−8.0	+13.0
	0.40	0.0689	−11.8	+21.7
	0.60	0.0728	−11.2	+8.1
	0.80	0.1017	−13.6	+9.0
	1.10	0.1473	−17.1	+8.2

**Table 3:** Influence of mesh density. Reinforcement stress at the member end at selected imposed-strain levels for the three models (ϕ20 mm).

Model	*ε_imp_* [‰]	Reinforcement Stress Reference [MPa]	Coarse Mesh Difference [%]	Fine Mesh Difference [%]
M1	0.20	84.6	−2.2	+1.4
	0.40	125.0	−2.4	+0.8
	0.60	147.8	+2.2	+0.2
	0.80	181.0	−0.7	+0.6
	1.13	239.0	−2.9	+0.7
M2	0.20	39.4	−5.1	+3.2
	0.40	37.2	−5.5	+3.8
	0.60	29.9	−5.5	+3.3
	0.80	25.6	−7.8	+5.1
	1.10	21.9	−10.5	+6.8
M3	0.20	82.8	+0.1	−1.8
	0.40	129.6	−0.5	+4.1
	0.60	168.0	+0.0	−0.3
	0.80	216.0	+0.1	+0.0
	1.10	286.0	+0.7	+0.7

**Table 4:** Influence of mesh density. Bond stress at the member end at selected imposed-strain levels for the three models (ϕ20 mm).

Model	*ε_imp_* [‰]	Bond Stress Reference [MPa]	Coarse Mesh Difference [%]	Fine Mesh Difference [%]
M1	0.20	3.270	+0.0	+0.3
	0.40	3.820	+0.0	+0.0
	0.60	2.538	+61.2	+2.0
	0.80	3.287	−2.6	+1.9
	1.13	4.160	−0.5	+0.0
M2	0.20	3.180	+0.0	+0.1
	0.40	3.726	−0.2	+0.1
	0.60	2.995	−0.3	+0.0
	0.80	3.803	−1.4	+0.3
	1.10	4.140	−0.5	+0.2
M3	0.20	3.054	+1.1	−5.5
	0.40	2.636	−0.8	+39.2
	0.60	3.340	+1.8	−5.7
	0.80	3.800	+0.0	−0.5
	1.10	4.090	+0.5	−0.2

**Table 5:** Crack widths in an element reinforced with ϕ10 rebar at the comparison strain level *ε_imp_* = 1.4‰.

Model	*w*_*cr*,*1*_	*w*_*cr*,*2*_	*w*_*cr*,*3*_	*w*_*cr*,*4*_	*w*_*m*,*cal*_	*w*_*lab*,*1*_	*w*_*lab*,*2*_	*w*_*lab*,*3*_	*w*_*m*,*lab*_	*δ*_w_ [%]
M1-ϕ10	0.328	0.314	0.344	0.196	0.296	0.34	0.31	0.32	0.32	−7.5
M2-ϕ10	0.302	0.289	0.315	0.170	0.269					−15.9
M3-ϕ10	0.312	0.278	0.337	0.247	0.294					−8.1

Note: All crack widths are expressed in mm. Relative differences are calculated using the rounded mean values reported in the table.

**Table 6:** Mean bond stresses |*τ_bm_*| [MPa] over half-final-spacing intervals truncated at zero bond stress; interval lengths [mm] are given in parentheses.

Number of Cracks	Cr5L	Cr5R	Cr1L	Cr1R	Cr7L	Cr7R	Cr3L	Cr3R	Cr8L	Cr8R	Cr4L	Cr4R	Cr2L	Cr2R	Cr6L	Cr6R	*τ_mean_*
1	—	—	1.40 (57.5)	1.68 (60.4)	—	—	—	—	—	—	—	—	—	—	—	—	1.54
2	—	—	2.06 (57.5)	2.25 (60.4)	—	—	—	—	—	—	—	—	1.84 (103.5)	2.15 (57.5)	—	—	2.08
3	—	—	2.49 (57.5)	2.41 (60.4)	—	—	2.59 (54.6)	2.75 (54.6)	—	—	—	—	2.20 (103.5)	2.57 (57.5)	—	—	2.50
4	—	—	3.44 (57.5)	3.42 (60.4)	—	—	3.48 (54.6)	3.37 (54.6)	—	—	3.59 (51.8)	2.59 (99.7)	2.48 (103.5)	3.50 (57.5)	—	—	3.24
5	2.18 (45.1)	1.95 (41.2)	1.52 (57.5)	3.46 (60.4)	—	—	3.50 (54.6)	3.40 (54.6)	—	—	3.62 (51.8)	2.62 (99.8)	2.51 (103.5)	3.51 (57.5)	—	—	2.83
6	2.38 (50.2)	2.02 (48.6)	1.59 (57.5)	3.63 (60.4)	—	—	3.72 (54.6)	3.65 (54.6)	—	—	3.85 (51.8)	2.85 (101.8)	2.68 (103.5)	1.59 (57.5)	2.01 (48.2)	2.35 (50.0)	2.69
7	2.62 (62.0)	2.41 (57.4)	1.86 (57.5)	1.97 (60.4)	2.43 (56.1)	2.07 (48.8)	1.90 (54.6)	4.05 (54.6)	—	—	4.24 (51.8)	3.24 (102.4)	3.13 (103.5)	2.02 (57.5)	2.40 (55.3)	2.60 (61.9)	2.64
8	2.66 (62.7)	2.47 (57.5)	1.92 (57.2)	2.01 (60.4)	2.46 (57.0)	2.12 (49.5)	1.95 (54.6)	1.76 (54.6)	1.99 (39.1)	1.79 (34.1)	1.80 (51.8)	3.26 (102.5)	3.16 (103.5)	2.07 (57.5)	2.44 (55.8)	2.63 (62.6)	2.28

Note: Cr denotes the crack number, and L and R denote the left-hand and right-hand sides, respectively.

**Table 7:** Bond coefficients *k*_1_ corresponding to Table 6, with *f_ctm_* = 2.13 MPa.

Number of Cracks	Cr5L	Cr5R	Cr1L	Cr1R	Cr7L	Cr7R	Cr3L	Cr3R	Cr8L	Cr8R	Cr4L	Cr4R	Cr2L	Cr2R	Cr6L	Cr6R	*k*_1,*mean*_
1	—	—	1.53	1.27	—	—	—	—	—	—	—	—	—	—	—	—	1.40
2	—	—	1.03	0.95	—	—	—	—	—	—	—	—	1.16	0.99	—	—	1.03
3	—	—	0.86	0.88	—	—	0.82	0.77	—	—	—	—	0.97	0.83	—	—	0.86
4	—	—	0.62	0.62	—	—	0.61	0.63	—	—	0.59	0.82	0.86	0.61	—	—	0.67
5	0.98	1.09	1.40	0.62	—	—	0.61	0.63	—	—	0.59	0.81	0.85	0.61	—	—	0.82
6	0.89	1.05	1.34	0.59	—	—	0.57	0.58	—	—	0.55	0.75	0.79	1.34	1.06	0.91	0.87
7	0.81	0.88	1.14	1.08	0.88	1.03	1.12	0.53	—	—	0.50	0.66	0.68	1.06	0.89	0.82	0.86
8	0.80	0.86	1.11	1.06	0.86	1.01	1.09	1.21	1.07	1.19	1.18	0.65	0.67	1.03	0.87	0.81	0.97

**Table 8:** Mean bond stresses |*τ_bm_*| [MPa] over stage-specific transfer lengths; transfer lengths [mm] are given in parentheses.

Number of Cracks	Cr5L	Cr5R	Cr1L	Cr1R	Cr7L	Cr7R	Cr3L	Cr3R	Cr8L	Cr8R	Cr4L	Cr4R	Cr2L	Cr2R	Cr6L	Cr6R	*τ_mean_*
1	—	—	0.94 (98.0)	0.43 (378.8)	—	—	—	—	—	—	—	—	—	—	—	—	0.68
2	—	—	1.35 (103.9)	1.12 (176.7)	—	—	—	—	—	—	—	—	0.83 (279.4)	1.40 (103.8)	—	—	1.18
3	—	—	1.65 (108.3)	1.52 (121.3)	—	—	1.70 (108.7)	1.50 (175.9)	—	—	—	—	1.43 (180.4)	1.70 (108.5)	—	—	1.58
4	—	—	2.45 (121.1)	2.50 (117.6)	—	—	2.57 (112.4)	2.41 (110.5)	—	—	2.67 (102.3)	2.59 (99.7)	2.40 (107.3)	2.55 (118.5)	—	—	2.52
5	2.18 (45.1)	1.95 (41.2)	1.26 (73.8)	2.54 (117.8)	—	—	2.60 (112.2)	2.44 (110.3)	—	—	2.69 (102.5)	2.62 (99.8)	2.42 (107.2)	2.50 (120.6)	—	—	2.32
6	2.38 (50.2)	2.02 (48.6)	1.41 (66.4)	2.74 (113.2)	—	—	2.71 (116.8)	2.69 (109.2)	—	—	2.91 (103.5)	2.85 (101.8)	2.64 (105.2)	1.40 (66.8)	2.01 (48.2)	2.35 (50.0)	2.34
7	2.62 (62.0)	2.41 (57.4)	1.86 (57.6)	1.85 (64.6)	2.43 (56.1)	2.07 (48.8)	1.73 (60.5)	3.12 (105.5)	—	—	3.17 (107.3)	3.24 (102.4)	3.09 (104.6)	1.95 (59.7)	2.40 (55.3)	2.60 (61.9)	2.47
8	2.66 (62.7)	2.46 (57.8)	1.92 (57.2)	1.91 (63.8)	2.46 (57.0)	2.12 (49.5)	1.80 (59.7)	1.46 (70.2)	1.99 (39.1)	1.79 (34.1)	1.47 (69.4)	3.26 (102.5)	3.13 (104.5)	2.01 (59.2)	2.44 (55.8)	2.63 (62.6)	2.22

Note: The same integration procedure was applied using the stage-specific transfer length *l_t_*.

**Table 9:** Bond coefficients *k*_1_ corresponding to Table 8, with *f_ctm_* = 2.13 MPa.

Number of Cracks	Cr5L	Cr5R	Cr1L	Cr1R	Cr7L	Cr7R	Cr3L	Cr3R	Cr8L	Cr8R	Cr4L	Cr4R	Cr2L	Cr2R	Cr6L	Cr6R	*k*_*1*,*mean*_
1	—	—	2.27	4.99	—	—	—	—	—	—	—	—	—	—	—	—	3.63
2	—	—	1.57	1.89	—	—	—	—	—	—	—	—	2.56	1.52	—	—	1.89
3	—	—	1.29	1.40	—	—	1.25	1.42	—	—	—	—	1.49	1.25	—	—	1.35
4	—	—	0.87	0.85	—	—	0.83	0.88	—	—	0.80	0.82	0.89	0.83	—	—	0.85
5	0.98	1.09	1.69	0.84	—	—	0.82	0.87	—	—	0.79	0.81	0.88	0.85	—	—	0.96
6	0.89	1.05	1.51	0.78	—	—	0.79	0.79	—	—	0.73	0.75	0.81	1.52	1.06	0.91	0.97
7	0.81	0.88	1.15	1.15	0.88	1.03	1.23	0.68	—	—	0.67	0.66	0.69	1.09	0.89	0.82	0.90
8	0.80	0.87	1.11	1.12	0.86	1.01	1.18	1.46	1.07	1.19	1.45	0.65	0.68	1.06	0.87	0.81	1.01

**Table 10:** Crack widths in an element reinforced with ϕ20 rebar at the comparison strain level *ε_imp_* = 1.1‰.

Model	*w*_*cr*,*1*_	*w*_*cr*,*2*_	*w*_*cr*,*3*_	*w*_*cr*,*4*_	*w*_*cr*,*5*_	*w*_*cr*,*6*_	*w*_*cr*,*7*_	*w*_*cr*,*8*_	*w*_*m*,*cal*_	*w*_*lab*,*1*_	*w*_*lab*,*2*_	*w*_*lab*,*3*_	*w*_*m*,*lab*_	*δ*_w_ [%]
M1-ϕ20	0.096	0.13	0.113	0.15	0.129	0.128	0.115	0.084	0.118	0.14	0.14	0.13	0.137	−13.9
M2-ϕ20	0.097	0.134	0.111	0.151	0.134	0.132	0.122	0.091	0.122					−10.9
M3-ϕ20	0.135	0.166	0.117	0.200	0.158	0.134	0.134	0.132	0.147					+7.3

Note: All crack widths are expressed in mm. Relative differences are calculated using the rounded mean values reported in the table.

**Table 11:** Quantitative comparison of cracking, reinforcement stresses and bond stresses for Models 1–3 at the final comparison levels.

Model	*ϕ*/*ρ* [mm]/[%]	*ε*_*imp*,*f*_ [‰]	*n_f_* [–]	*w*_*m*,*f*_ [mm]	*σ*_*s*,*max*,*f*_ [MPa]	$\sigma_{s,f}^{-}$ [MPa]	*σ*_*s*,*peak*_ [MPa]	*τ*_*b*,*max*,*f*_ [MPa]
M1	10/0.785	1.40	4	0.296	414	306	414	5.8
M2	10/0.785	1.40	4	0.269	95	0	218	4.8
M3	10/0.785	1.40	4	0.294	448	334	448	5.6
M1	20/3.14	1.10	8	0.118	243	213	243	4.6
M2	20/3.14	1.10	8	0.122	23.8	0	43.6	4.6
M3	20/3.14	1.10	8	0.147	286	263	286	4.1

Note: The comparison strain follows the model-specific definitions in Section 2. The signed spatial mean reinforcement stress was obtained by element-wise integration over the full restrained bar length of 1150 mm, divided by that length.

## Data Availability

The original contributions presented in this study are included in the article. Further inquiries can be directed to the corresponding author.

## Abbreviations

Symbols	
*A_c_*, *A_s_*	cross-sectional areas of concrete and reinforcement, respectively
*C*	coefficient in the power-law bond–slip relation
*d_c_*	distance from the concrete surface considered to the centre of the nearest reinforcing bar
*E_c_*	elastic modulus of concrete used in the analytical relations; taken as *E_ct_* specified in Table 1
*E_ct_*	Young’s modulus of concrete in tension
*E_s_*	Young’s modulus of reinforcement
*f_cm_*	mean cylinder compressive strength of concrete
*f_c_*_,*cube*_	cube compressive strength of concrete used in the bond–slip law
*f_ctm_*	mean tensile strength of concrete
*G_f_*	fracture energy of concrete
*k_1_*	local bond coefficient, *k_1_* = *f_ctm_*/|*τ_bm_*|
*k_1_*_,*mean*_	unweighted arithmetic mean of the local bond coefficients over all crack sides present at the considered cracking stage
*l*	length of the integration interval used to evaluate the local mean bond stress
*l_t_*	stage-specific transfer length from a crack to the first zero crossing of bond stress on the considered side
*L*	member length
*N*	total axial force resulting from the end reactions; tension positive
*N_cr_*	cracking force
*N_c_(x)*	axial force carried by concrete at position *x*; tension positive
*N_s_(x)*	axial force carried by reinforcement at position *x*; tension positive
Δ*N*(*x*)	self-equilibrated axial-force component, acting with opposite signs in concrete and reinforcement
Δ*T*	uniform temperature change used in the analytical thermal relations; negative for cooling
Δ*T_c_*	change in the calculated, approximately uniform concrete temperature relative to its initial value
*n_f_*	number of cracks at the final comparison level
*s*	local slip at the steel–concrete interface
*u_s_*(*x*)	axial displacement of reinforcement at position *x*
*w*_*cr*,*i*_	calculated width of crack *i*
*w*_*lab*,*j*_	mean measured surface crack width considering all cracks in experimental specimen *j*
*w_m_*_,*f*_	mean calculated surface crack width at the final comparison level
*w*_*m*,*cal*_	mean calculated width of all cracks at the considered deformation level
*w*_*m*,*lab*_	arithmetic mean of the mean measured crack widths for the three experimental specimens
*x*	longitudinal coordinate along the member
*α*	exponent in the bond–slip relation
*α_c_*, *α_s_*	coefficients of thermal expansion of concrete and reinforcement, respectively
*δ_w_*	signed relative difference between calculated and experimental mean crack widths, defined by Equation (9)
*ε*	axial strain
*ε_c_*, *ε_s_*	total axial strains of concrete and reinforcement, respectively
*ε_cm_*	mean concrete strain
*ε_imp_*	model-specific comparison strain: resulting mean concrete gauge strain in M1, magnitude of the prescribed concrete eigenstrain in M2, and magnitude of the nominal free concrete thermal strain in M3
*ε*_*imp*,*f*_	model-specific comparison strain at the final comparison level
*ε_sm_*	mean reinforcement strain
*ε_sh_*	prescribed longitudinal shrinkage-type eigenstrain of concrete; negative for contraction
*ν_c_*, *ν_s_*	Poisson’s ratios of concrete and reinforcement, respectively
*σ_c_*, *σ_s_*	axial stresses in concrete and reinforcement, respectively; tension positive
*σ*_*s*,*max*,*f*_	maximum tensile reinforcement stress at the final comparison level
$\sigma_{s,f}^{-}$	signed spatial average of reinforcement stress over the full restrained length at the final comparison level
*σ*_*s*,*peak*_	maximum tensile reinforcement stress over all positions and analysis steps up to the final comparison level
*ρ*	reinforcement ratio
*τ*(*s*)	bond stress as a function of slip
*τ*(*x*)	signed bond stress at longitudinal position x
|*τ_bm_*|	absolute value of the signed mean bond stress over the considered integration interval
*τ_mean_*	unweighted arithmetic mean of the local absolute mean bond stresses over all crack sides present at the considered cracking stage
*τ_b_*_,*max*,*f*_	maximum absolute bond stress at the final comparison level
ϕ	bar diameter
Subscripts	
*b*	bond
*c*	concrete
*cal*	calculated
*cr*	crack or cracking
*eff*	effective
*f*	final comparison level
*i*	numerical crack index
*imp*	imposed
*j*	experimental specimen index
*lab*	laboratory
*m*	mean
*max*	maximum
*mean*	arithmetic mean over crack sides
*peak*	maximum over the analysed loading history
*s*	steel
*sh*	shrinkage
*t*	tension in *E_ct_* and transfer in *l_t_*.
2D	two-dimensional
ACI	American Concrete Institute
CEN	European Committee for Standardization
Cr	numerical crack identifier; the number denotes the order of crack formation
DIANA	finite-element analysis software used in this study
EXX	total normal strain in the x-direction (DIANA output label)
FE	finite element
fib	International Federation for Structural Concrete
L, R	left-hand and right-hand sides of a crack, respectively, in crack labels
LF	load factor
M1, M2, M3	imposed-strain Models 1, 2 and 3, respectively
RC	reinforced concrete
SLS	serviceability limit state
SXX	Cauchy normal stress in the x-direction (DIANA output label)
